# A doubly robust estimator for continuous treatments in high dimensions

**DOI:** 10.1186/s12874-025-02488-3

**Published:** 2025-02-13

**Authors:** Qian Gao, Jiale Wang, Ruiling Fang, Hongwei Sun, Tong Wang

**Affiliations:** 1 Department of Health Statistics, School of Public Health, MOE Key Laboratory of Coal Environmental Pathogenicity and Prevention, Shanxi Medical University, No.56 Xinjian South Road, Taiyuan, 030001 China; 2 Department of Health Statistics, School of Public Health, Binzhou Medical University, Yantai, China

**Keywords:** Causal inference, Doubly robust, High-dimensional data, Generalized propensity score

## Abstract

**Background:**

Generalized propensity score (GPS) methods have become popular for estimating causal relationships between a continuous treatment and an outcome in observational studies with rich covariate information. The presence of rich covariates enhances the plausibility of the unconfoundedness assumption. Nonetheless, it is also crucial to ensure the correct specification of both marginal and conditional treatment distributions, beyond the assumption of unconfoundedness.

**Method:**

We address limitations in existing GPS methods by extending balance-based approaches to high dimensions and introducing the Generalized Outcome-Adaptive LASSO and Doubly Robust Estimate (GOALDeR). This novel approach integrates a balance-based method that is robust to the misspecification of distributions required for GPS methods, a doubly robust estimator that is robust to the misspecification of models, and a variable selection technique for causal inference that ensures an unbiased and statistically efficient estimation.

**Results:**

Simulation studies showed that GOALDeR was able to generate nearly unbiased estimates when either the GPS model or the outcome model was correctly specified. Notably, GOALDeR demonstrated greater precision and accuracy compared to existing methods and was slightly affected by the covariate correlation structure and ratio of sample size to covariate dimension. Real data analysis revealed no statistically significant dose-response relationship between epigenetic age acceleration and Alzheimer’s disease.

**Conclusion:**

In this study, we proposed GOALDeR as an advanced GPS method for causal inference in high dimensions, and empirically demonstrated that GOALDeR is doubly robust, with improved accuracy and precision compared to existing methods. The R package is available at https://github.com/QianGao-SXMU/GOALDeR.

**Supplementary Information:**

The online version contains supplementary material available at 10.1186/s12874-025-02488-3.

## Introduction

The advent of omics data and health care data makes it possible to draw causal conclusions from observational studies because a substantial number of covariates makes the assumption of unconfoundedness plausible [1]. The propensity score (PS) method is a common statistical tool for performing such causal inference in observational studies. The PS method was originally developed to estimate the causal effects of a binary treatment, exposure, or intervention (hereafter referred to as ‘treatment’) on an outcome [2]. Recently, extensions of PS methods to the context of continuous treatment have been developed and are collectively known as generalized PS (GPS) methods. GPS methods are focused on estimating the dose–response function (DRF) describing the relationship between a continuous treatment and an outcome [3–5]. Similar to PS methods, GPS methods estimate the DRF through regression adjustment [5], matching [6], stratification [7], and inverse probability weighting (IPW) [8]. Additionally, the doubly robust approach has been proposed and has received increasing attention as a robust method to model misspecification of either the GPS model or the outcome model [9, 10].

GPS is a probability density function of the treatment conditional on observed covariates [5]. The validity of GPS methods relies on the assumption that both the conditional mean and the conditional distribution of the treatment, given the covariates, must be correctly specified [8]. To relax this assumption, several balancing approaches have been proposed under a weighting or doubly robust framework [11–15]. The balancing approaches are focused on directly estimating weights under the balance constraints, including covariate balance; specifically, the weighted cross-moments between the treatment and each covariate are 0. Recent methods include the nonparametric covariate balancing generalized propensity score (npCBGPS) of Fong et al. [11], entropy balancing weights [12, 13], and covariate association eliminating weights of Yiu et al. [14]. Whereas these methods are appealing in terms of robustness to GPS model misspecification, the orders of the moment of both the covariates and the treatment to decorrelate must be carefully chosen. A higher moment may be helpful when there are nonlinear correlations between the treatment and the covariates, but this may violate the positivity assumption [13, 15]. To our knowledge, there is still a lack of guidance for specifying correct orders of moment, which is necessary to mitigate confounding bias. To address the issue of what moments to decorrelate, Huling et al. proposed distance covariance optimal weights (DCOWs) [15]. However, the abovementioned methods do not consider variable selection, which is another important factor influencing the performance of the estimated DRF [16–21]; therefore, their application is limited in the case of high-dimensional covariates.

The GPS methods are susceptible to the covariates being balanced. For example, the inclusion of instrumental variables (IVs) that can only predict the treatment in the GPS model could inflate variance without reducing bias in the estimates [16–21]. It has been well documented that an optimal GPS method should balance or control for all confounders and prognostic covariates that can only predict the outcome [16–21]. Doing so can not only remove confounding bias but also improve the efficiency of the estimates [16–21]. Hence, it is necessary to introduce variable selection techniques into GPS methods in high-dimensional context. In the doubly robust framework, Su et al. [22] and Colangelo et al. [23] used machine learning methods, and Antonelli et al. [24] used a Gaussian process to estimate nuisance parameters related to the GPS model and the outcome model. Unfortunately, these studies failed to address the adverse influence of IVs. Under adaptive lasso-based shrinkage, our previously proposed generalized outcome-adaptive LASSO (GOAL) approach discourages the selection of IVs by strongly penalizing covariates that are not associated with the outcome [25]. The GOAL method is robust to the GPS model misspecification. However, its validity depends on the assumption that the outcome model is linear.

Here, we retained the idea of variable selection from the GOAL method and proposed a generalized outcome-adaptive LASSO and doubly robust estimation (GOALDeR) method. Unlike the GOAL method, our proposed method constructs a penalty function that is independent of the outcome model. Consequently, we can estimate the DRF in the doubly robust framework [21]. In recognizing that the correlation between the treatment and confounders is a source of confounding bias [15], our method uses a distance correlation coefficient as a measure to assess covariate balance. The distance correlation coefficient is zero if and only if the variables are independent of each other [26]. With a simulation, we show that the GOALDeR method is doubly robust, provides more precise and accurate estimates than existing methods, and is scarcely affected by the covariate correlation structure and ratio of sample size to covariate dimension. We also applied the GOALDeR method to investigate potential causality between epigenetic age acceleration and Alzheimer’s disease (AD).

## Generalized outcome-adaptive LASSO and doubly robust estimation

### Notations and assumptions

We let $D_{{i=1}}^{n}=\left( {{T_i},{Y_i},{Z_i}} \right)$ denotes an independent and identically distributed sample drawn from a common joint distribution $f\left( {T,Y,Z} \right)$. Each subject $i \in \left\{ {1,...,n} \right\}$ has a continuous treatment ${T_i}$ whose support is $\:\mathcal{T}\subseteq\:\mathcal{R}$, and an outcome $\:{Y}_{i}$. We characterize causal DRF using potential outcome notation [27] and define $\:{Y}_{i}\left(t\right)$ as the potential outcome for subject $i \in \left\{ {1,...,n} \right\}$ given treatment level $\:{T}_{i}=t$ ($\:t\in\:\mathcal{T}$). Our target estimand is $\:\mathbb{E}\left({Y}_{i}\left(t\right)\right)$. The observed $\:{\varvec{Z}}_{i}\in\:{\mathcal{R}}^{p}$ denotes pre-treatment covariates, where *p* is the dimension. Each available covariate belongs to one of four mutually exclusive covariate sets:

- confounders ($\:{\varvec{Z}}_{c}$): covariates that contribute to both the treatment and the outcome;
- prognostic covariates ($\:{\varvec{Z}}_{P}$): covariates that contribute to the outcome only;
- instrumental variables ($\:{\varvec{Z}}_{I}$): covariates that contribute to the treatment only;
- spurious covariates ($\:{\varvec{Z}}_{S}$): covariates that contributions to neither the treatment nor the outcome.

Under the potential outcome framework, we established the following assumptions to identify the DRF from the observed data, and we maintained these assumptions throughout this work.

Assumption 1 **(Consistency)**: For subject $i \in \left\{ {1,\cdots,n} \right\}$, $T_{i} = (t \in\mathcal{T})$ implies $Y_{i} = Y_{i}(\text{t})$.

Assumption 2 **(Positivity)**: The GPS or conditional probability density function of the treatment $f_{\text{T|Z}} (T_{\text{i}}= t|Z_{\text{i}})$ is positive for any $t \in \mathcal{T}$ and for any ${\varvec{Z}}_{i}\in\:{\mathcal{R}}^{p}$.

Assumption 3 **(Unconfoundedness)**: $Y_{\text{i}}(\text{t})\perp T_{\text{i}} | Z_{\text{i}}, \forall \:t\in\:\mathcal{T}$ means that for any treatment level, the potential outcome $Y_{\text{i}}(\text{t})$ is conditionally independent of the treatment given the covariates. Note that this assumption is untestable from the observed data.

Assumption 4 **(Stable unit treatment assumption)**: This assumption indicates that there is no interference among subjects.

### Variable selection based on outcome-adaptive LASSO

We retained the idea of variable selection from the GOAL method [25] and started by assuming the GPS model as follows:

1 $$\:E\left(T|\varvec{Z}\right)={\alpha\:}_{0}+\sum\:_{j=1}^{p}{Z}_{j}{\alpha\:}_{j}$$

As mentioned in the introduction, an optimal GPS method should control for or balance covariates that are associated with the outcome (including $\:{\varvec{Z}}_{c}$ and $\:{\varvec{Z}}_{P}$). The covariate selection mechanism should be free from the outcome model for a doubly robust estimator. We borrowed the idea from the adaptive LASSO and achieved a covariate selection procedure by solving:

2 $$\:\widehat{\alpha\:}=arg\ \underset{\alpha\:}{\text{min}}{||T-{\alpha\:}_{0}-\sum\:_{j=1}^{p}{Z}_{j}{\alpha\:}_{j}\|}_{2}^{2}+{\lambda\:}_{n}\sum\:_{j=1}^{p}{\widehat{w}}_{j}\left|{\alpha\:}_{j}\right|$$

where $\:{\widehat{w}}_{j}$ denotes penalty weight and is inversely proportional to the influence of covariates $\:{Z}_{j}$ on the outcome. Here, the GOALDeR method defines an outcome model-free penalty weight as $\:{\widehat{w}}_{j}={\left|\left|dcor\left({Z}_{j},Y|T\right)\right|/\underset{j}{\text{max}}\left|dcor\left({Z}_{j},Y|T\right)\right|\right|}^{\gamma\:}$, where $\:dcor\left({Z}_{j},Y|T\right)$ is the conditional distance correlation coefficient between $\:{Z}_{j}$ and the outcome $\:Y$, given treatment $\:T$, measuring any kind of correlations [28]. $\:\gamma\:>1$ is a tuning parameter. $\:{\lambda\:}_{n}>0$ is another tuning parameter satisfying $\:{{\uplambda\:}}_{\text{n}}/\sqrt{\text{n}}\to\:0$ and $\:{{\uplambda\:}}_{\text{n}}{\text{n}}^{{\upgamma\:}/2-1}\to\:{\infty\:}$ for consistency in variable selection, as with the GOAL method [25, 29]. On the contrary, the GOAL method utilizes coefficients from a separate linear outcome model to create penalty weights, which means that the validity of the GOAL method depends on the correct specification of the outcome model.

#### Choosing ${\varvec{\lambda}}_{\varvec{n}}$ 

We propose dual-weight distance correlation (DWDC) as a measure for selecting the optimal $\:{\lambda\:}_{n}$, and the rule is minimizing DWDC. Similar to dual-weight correlation (DWC) in the GOAL method, the standpoint of DWDC is covariate balance for unbiased, efficient estimation. However, unlike DWC which only captures linear correlations between covariates and both the treatment and the outcome, DWDC uses distance correlation to capture all types of correlations between covariates and both the treatment and the outcome.

3 $$\:DWDC=\sum\:_{j=1}^{p}{\left|dcor\left({Z}_{j},Y|T\right)\right|}^{2}\left|{dcor}_{{w}^{{\lambda\:}_{n}}}\left({Z}_{j},T\right)\right|$$

where $\:{dcor}_{{w}^{{\lambda\:}_{n}}}\left({Z}_{j},T\right)$ refers to the weighted distance correlation between covariate $\:{Z}_{j}$ and the treatment, serving as a measure of covariate balance. The smaller the $\:\left|{dcor}_{{w}^{{\lambda\:}_{n}}}\left({Z}_{j},T\right)\right|$, the better the covariate balance achieved after weighting. Recall that $\:dcor\left({Z}_{j},Y|T\right)$ is the conditional distance correlation between $\:{Z}_{j}$ and $\:Y$ given *T*. Multiplying these two components implies that the DWDC is more affected by the imbalance of $\:{\varvec{Z}}_{c}$ and $\:{\varvec{Z}}_{P}$ and less affected by the imbalance of $\:{\varvec{Z}}_{I}$ and $\:{\varvec{Z}}_{S}$. Hence, a smaller DWDC could further encourage the selection of $\:{\varvec{Z}}_{c}$ and $\:{\varvec{Z}}_{P}$.

The balance weights $\:{w}^{{\lambda\:}_{n}}$ in the DWDC are estimated using the DCOWs method with covariates selected according to Eq. (2) with $\:{\lambda\:}_{n}$, without requiring the specification of moment orders for both the covariates and the treatment to achieve decorrelation. The DCOWs method uses weighted distance covariance between the treatment and covariates as a loss function and directly estimates balance weights under the following constraints: (1) the marginal distributions of the treatment and the covariates are preserved after weighting; (2) the weights are positive and sum to the sample size. The authors showed that the balance weights estimated by the DCOWs could enhance a doubly robust estimator. Further details are provided in the article by Huling et al. [15]. On the contrary, the balance weights $\:{w}^{{\lambda\:}_{n}}$ in the DWC are estimated using npCBGPS [11], which requires the specification of moment orders for both the covariates and the treatment to achieve decorrelation of nonlinearities.

### Estimating DRF using a doubly robust estimator

Based on balance weights estimated using covariates selected by optimal $\:{\lambda\:}_{n}$, the GOAL method uses the IPW method to estimate DRF. The GOAL method cannot estimate DRF using the “doubly robust” method as variable selection in the GPS model hinges on the outcome model being correct, which undermines the “doubly robust” nature of the method [21]. In contrast, the variable selection in GOALDeR is independent of the outcome model; therefore, we ultimately use the doubly robust estimator of Kennedy et al. [10] to estimate DRF. The doubly robust estimator of Kennedy et al. [10] consists of two steps. In the first step, a pseudo-outcome is constructed, and in the second step, the pseudo-outcome is regressed on the treatment to estimate DRF. The pseudo-outcome can be estimated as [15]:

4 $$\:\widehat{\theta\:}\left({T}_{i}\right)=\frac{1}{n}\sum\:_{i=1}^{n}\widehat{\mu\:}\left(\stackrel{-}{\varvec{Z},}{T}_{i}\right)+\left({Y}_{i}-\widehat{\mu\:}\left({\varvec{Z}}_{i},{T}_{i}\right)\right){w}_{i}$$

where $\:\widehat{\mu\:}\left(\varvec{Z},T\right)$ denotes an estimate of the outcome model $\:\mu\:\left(\varvec{Z},T\right)$. Here, the Super Learner method (SL) which combines LASSO, XGBoost, Random Forest, and Support vector machines is applied to estimate $\:\widehat{\mu\:}\left(\bullet\:\right)$ [30]. $\:{w}_{i}$ denotes balance weights estimated by the DCOWs method with covariates selected by optimal $\:{\lambda\:}_{n}$. Subsequently, the DRF is estimated using a linear or nonlinear regression model of the treatment on the pseudo-outcome. In this work, we used a linear regression model for comparison purposes.

## Simulations

### Simulation setup

We modeled simulations to assess the performance of GOALDeR and compare it with existing approaches when there are a large number of covariates. Following our previous studies [25, 31], we developed simulations by adapting the research conducted by Tan et al. [32] and Shortreed et al. [29]. For each replicated dataset, *p* covariates and *n* individuals were drawn independently from a multivariate standard Gaussian distribution with varying correlations of 0, 0.2, and 0.5. We generated a continuous treatment and outcome from models given by:

5 $$\:\text{G}\text{P}\text{S}\:\text{m}\text{o}\text{d}\text{e}\text{l}:\:T=\sum\:_{j=1}^{p}m\left({Z}_{j}\right){\alpha\:}_{j}+\zeta\:,\:\zeta\:\sim N\left(\text{0,1}\right)$$

6 $$\:\text{O}\text{u}\text{t}\text{c}\text{o}\text{m}\text{e}\:\text{m}\text{o}\text{d}\text{e}\text{l}:\:Y=\eta\:T+\sum\:_{j=1}^{p}g\left({Z}_{j}\right){\beta\:}_{j}+\xi\:,\:\xi\:\sim N\left(\text{0,1}\right)$$

where $$\:\eta\:=0$$ or 2.

We used two data-generating scenarios to compare the GOALDeR method with existing methods, which were summarized in Table 1. In the first scenario, we assumed that both the GPS model and the outcome model are linear, that is, $\:g\left({Z}_{j}\right)={Z}_{j}$ and $\:m\left({Z}_{j}\right)={Z}_{j}$, $\:j=1,\dots\:p$, and we conducted simulations in three settings by varying the strength of the relationship between confounders and outcome, and treatment. We considered varying levels of confounding because the strength of the confounders affects the bias, variance, and mean-squared error (MSE) of an estimate [19]. For all three settings, the first two covariates, $\:{Z}_{1}$ and $\:{Z}_{2}$, are true confounders; the third and fourth covariates, $\:{Z}_{3}$ and $\:{Z}_{4}$, are prognostic covariates; the fifth and sixth covariates, $\:{Z}_{5}$ and $\:{Z}_{6}$, are IVs; and the other *p-6* covariates are spurious covariates. The first setting (SoSt) sets $\:\varvec{\alpha\:}=\left(\text{1,1},\text{0,0},\text{1,1},0,\dots\:\dots\:,0\right)$ and $\:\varvec{\beta\:}=\left(\text{1,1},\text{1,1},\text{0,0},0,\dots\:\dots\:,0\right)$. The second setting (SoWt) sets $\:\varvec{\alpha\:}=\left(\text{0.5,0.5,0},\text{0,1},\text{1,0},\dots\:\dots\:,0\right)$ and $\:\varvec{\beta\:}=\left(\text{1,1},\text{1,1},\text{0,0},0,\dots\:\dots\:,0\right)$. The third setting (WoSt) sets $\:\varvec{\alpha\:}=\left(\text{1,1},\text{0,0},\text{1,1},0,\dots\:\dots\:,0\right)$ and $\:\varvec{\beta\:}=\left(\text{0.5,0.5,1},\text{1,0},\text{0,0},\dots\:\dots\:,0\right)$. The coefficients of 1 and 0.5 for confounders are commonly used in epidemiology [31, 33–35].

Under the second scenario, we introduced model misspecification via a nonlinear transformation of confounders and conducted simulations under three settings by varying whether the GPS model or the outcome model was misspecified. The data-generating processes were similar to those in the simulations by Tan et al. [32] and Kang et al. [36], which explored the impact of model misspecification on DR and non-DR estimators. The first setting correctly specified the outcome model, and misspecified the GPS model (CoMt) given $\:m\left({Z}_{1}\right)=exp\left({Z}_{1}/2\right)$, $\:m\left({Z}_{2}\right)=\left({Z}_{2}/\left(1+exp\left({Z}_{1}\right)\right)\right)+10$, $\:m\left({Z}_{3}\right)={\left(0.04*{Z}_{1}*{Z}_{3}+0.6\right)}^{3}$, $\:m\left({Z}_{4}\right)={\left({Z}_{2}+{Z}_{4}\right)}^{2}$, $\:m\left({Z}_{j}\right)={Z}_{j}$ for $\:j>4$, and $\:g\left({Z}_{j}\right)={Z}_{j}$ for $\:j=1,\dots\:p$. The second setting (MoCt) used a nonlinear data-generating process for the outcome, and linear for the treatment given $\:m\left({Z}_{j}\right)={Z}_{j}$ for $\:j=1,\dots\:p$, and $\:g\left({Z}_{1}\right)=exp\left({Z}_{1}/2\right)$, $\:g\left({Z}_{2}\right)=\left({Z}_{2}/\left(1+exp\left({Z}_{1}\right)\right)\right)+10$, $\:g\left({Z}_{3}\right)={\left(0.04*{Z}_{1}*{Z}_{3}+0.6\right)}^{3}$, $\:g\left({Z}_{4}\right)={\left({Z}_{2}+{Z}_{4}\right)}^{2}$, $\:g\left({Z}_{j}\right)={Z}_{j}$ for $\:j>4$. The third setting (MoMt) used a nonlinear data-generating process for both the outcome and treatment, as with CoMt and MoCt. For all three settings, the coefficients were set to $\:\varvec{\alpha\:}=\left(\text{1,1},\text{1,1},\text{0,0},\text{1,1},0,\dots\:\dots\:,0\right)$ and $\:\varvec{\beta\:}=\left(\text{1,1},\text{1,1},\text{1,1},\text{0,0},0,\dots\:\dots\:,0\right)$.

For other settings, including the true causal parameter in the DRF ($\:\eta\:=0$ or 2), sample size, and the dimension of covariates, we followed Shortreed et al. [29] and our previous study [25]. For each setting, we generated 100 simulated datasets each for dimensionality (*n*/*p* ratio): *n* = 200, *p* = 100 and *n* = 500, *p* = 200. We searched over several possible $\:{\lambda\:}_{n}$ values $\:\left\{{n}^{-10},{{n}^{-5},{n}^{-2},{n}^{-1.25},{n}^{-1},n}^{-0.75},{n}^{-0.5},{n}^{-0.25},{n}^{0.25},{n}^{0.49}\right\}$ for each dataset and chose $\:\gamma\:$ such that $\:{{\uplambda\:}}_{\text{n}}{\text{n}}^{{\upgamma\:}/2-1}={n}^{2}$.

**Table 1:** Simulation scenarios. Treatment *T* is generated as $\:N\left(m\left(\varvec{Z}\right),1\right)$, and outcome *Y* is generated as $\:N\left(\eta\:T+g\left(\varvec{Z}\right),1\right)$ where $\:\eta\:=0\:or\:2$

Scenarios	Covariates (***Z***)	(*n*, *p*)	Settings	$\:m\left(\varvec{Z}\right)$ (treatment)	$\:g\left(Z\right)$ (outcome)
1	$Z_p\sim N\left({\mathbf0}_{p,}\mathrm\Sigma\right)$ $\:{{\Sigma\:}}_{ij}=1\:(i=j$) $\:{{\Sigma\:}}_{ij}=\rho\:(i\ne\:j$) $\rho=\;0,0.2,0.5$	(200,100) (500,200)	SoSt	$Z_1+Z_2+Z_5+Z_6$	$Z_1+Z_2+Z_3+Z_4$
			SoWt	$0.5\;\ast\:Z_1+0.5\;\ast\:Z_2\;+\;Z_5\;+\;Z_6$	$Z_1+Z_2+Z_3+Z_4$
			WoSt	$Z_1+Z_2+Z_5+Z_6$	$0.5\;\ast\:Z_1+0.5\;\ast\;Z_2+Z_3+Z_4$
2	$Z_p\sim N\left({\mathbf0}_{p,}\mathrm\Sigma\right)$ $\:{{\Sigma\:}}_{ij}=1\:(i=j$) $\:{{\Sigma\:}}_{ij}=\rho\:(i\ne\:j$) $\rho=\;0,0.2.0.5$	(200,100) (500,200)	CoMt	$$\begin{array}{l} [exp (Z_{1}/2)]\\ + [(Z_{2}/(1+ exp(Z_{1}))) + 10] \\ + [(0.04 \ast Z_{1} \ast Z_{3} + 0.6)^{3}] \\ + [(Z_{2} + Z_{4})^{2}] + Z_{7} + Z_{8} \end{array}$$	$Z_1+Z_2+Z_3+Z_4+Z_5+Z_6$
			MoCt	$Z_1+\;Z_2+Z_3+Z_4+Z_7+Z_8$	$$\begin{array}{l} [exp (Z_{1}/2)]\\ + [(Z_{2}/(1+ exp(Z_{1}))) + 10] \\ + [(0.04 \ast Z_{1} \ast Z_{3} + 0.6)^{3}] \\ + [(Z_{2} + Z_{4})^{2}] + Z_{5} + Z_{6} \end{array}$$
			MoMt	$$\begin{array}{l} [exp (Z_{1}/2)]\\ + [(Z_{2}/(1+ exp(Z_{1}))) + 10] \\ + [(0.04 \ast Z_{1} \ast Z_{3} + 0.6)^{3}] \\ + [(Z_{2} + Z_{4})^{2}] + Z_{7} + Z_{8} \end{array}$$	$$\begin{array}{l} [exp (Z_{1}/2)]\\ + [(Z_{2}/(1+ exp(Z_{1}))) + 10] \\ + [(0.04 \ast Z_{1} \ast Z_{3} + 0.6)^{3}] \\ + [(Z_{2} + Z_{4})^{2}] + Z_{5} + Z_{6} \end{array}$$

Furthermore, to investigate the impact of effect size on statistical testing, we also explored the performance of each method when the DRF parameter was set to 0.4 and 0.7. The data-generating processes are the same as those described in Table 1, with the only difference being η = 0.4 or 0.7. To examine the performance of the GOALDeR method as the sample size increases, we let *p* = 20 and *n* = 200, 500, 1000. The data-generating processes are the same as those described in Table 1, with the only difference being the values of (*n*,* p*).

### Comparing methods

 We compared the following methods for estimating DRF: (1) GOAL [25], whose processes are similar to those of the GOALDeR method. The main differences between the GOAL method and the GOALDeR method are described in Sect. 2. A detailed implementation of the GOAL method can be found in the Supplementary Materials; (2) SL-DR, which estimates the DRF in the DR framework of Kennedy et al. [10] (described in subsection 2.4). Briefly, the SL-DR method fits the GPS model and the outcome model using the SL method to estimate the pseudo-outcome. The SL method combines the results of LASSO, XGBoost, Random Forest, and Support vector machines. The balance weights used to estimate the pseudo-outcome are given by $\:{w}_{i}={f}_{T}\left({T}_{i}\right)/{f}_{T|Z}\left({T}_{i}|{\varvec{Z}}_{\varvec{i}}\right)$ where the numerator is the marginal density of the treatment, and $\:{f}_{T|Z}\left({T}_{i}|{\varvec{Z}}_{\varvec{i}}\right)$ is the GPS. In this study, we normally approximated both $\:{f}_{T}\left({T}_{i}\right)$ and $\:{f}_{T|Z}\left({T}_{i}|{\varvec{Z}}_{\varvec{i}}\right)$. The R packages used to implement the GOALDeR, SL-DR, and GOAL methods are available at https://github.com/QianGao-SXMU/GOALDeR and https://github.com/QianGao-SXMU/GOAL, respectively.

## Results

The results of data-generating with $\:\eta\:=2$ are shown following. The others are in the Supplementary Materials.

### Estimation under scenarios 1 and 2 with a modest *p* = 20

**Fig. 1:**
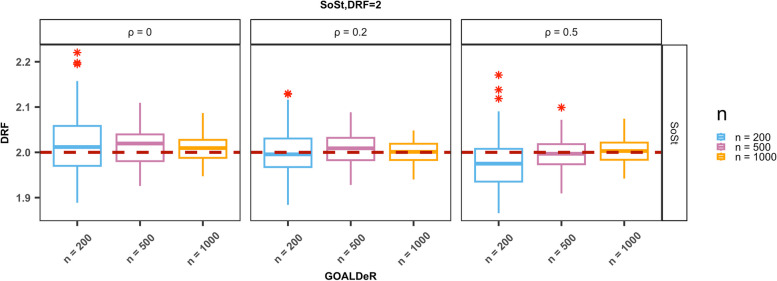
Illustrations with a modest *p* = 20. Boxplot of parameters for the dose–response function (DRF) under the setting where confounders are strongly correlated with both the treatment and outcome (SoSt) and η = 2. The true causal parameter of 2 is indicated by a dotted line, and the asterisks represent outliers

**Fig. 2:**
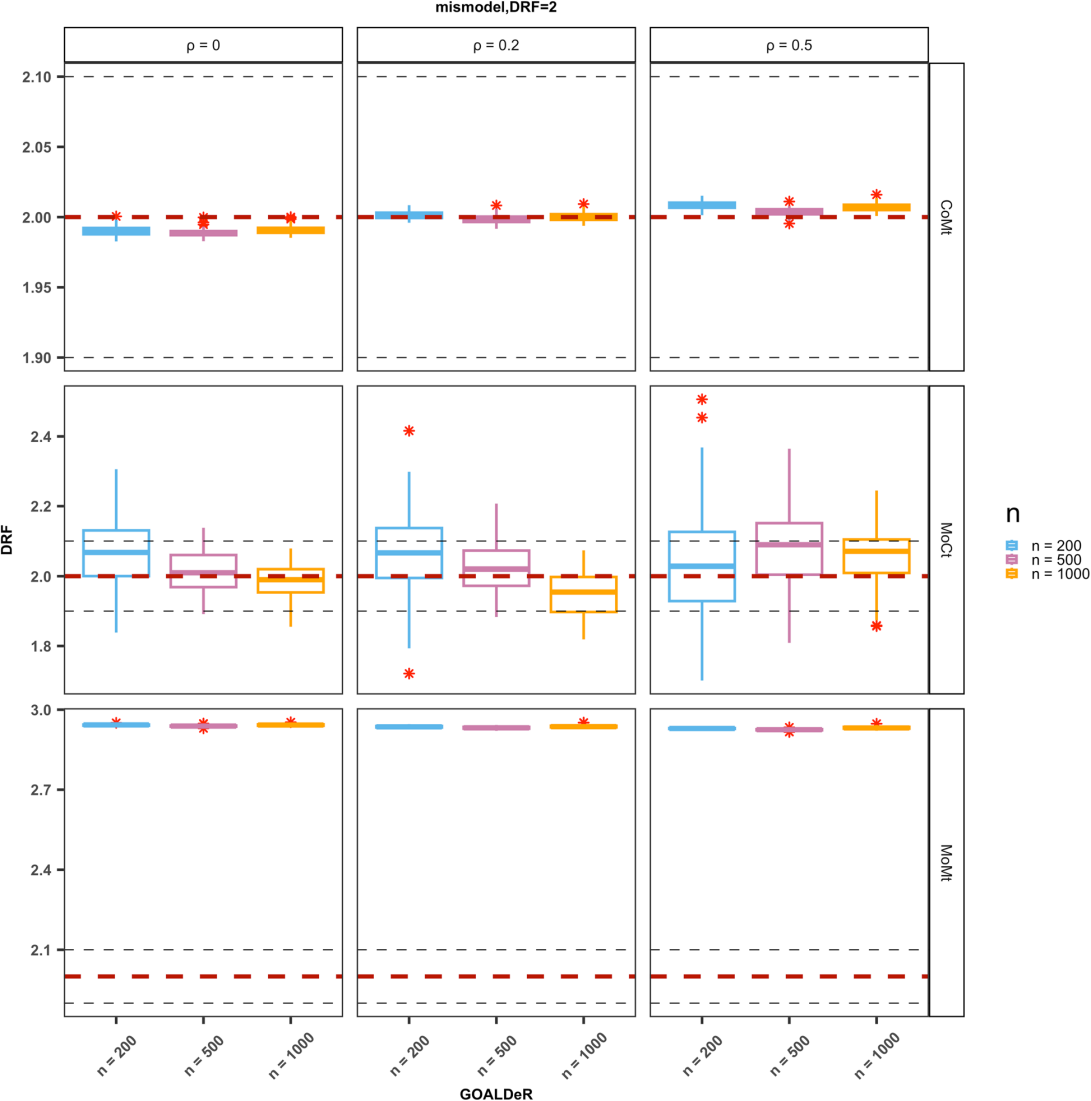
Illustrations with a modest *p* = 20. Boxplot of parameters for the dose–response function (DRF) under Scenario 2 with η = 2. The true causal parameter of 2 is indicated by a red dotted line, and the asterisks represent outliers. The black dashed lines at 1.9 and 2.1 are primarily intended to aid in evaluation

We performed simulations to evaluate GOALDeR and compare it with existing methods. For illustrating the performance of GOALDeR as the sample size (*n*) increases, we plotted the distribution of the causal parameter estimates using a boxplot and the proportion of times each covariate was selected for simulation with a modest number of covariates (*p* = 20). For Scenario 1, where both the outcome and GPS models are linear, we present the results for SoSt (the confounders are strongly correlated with both the treatment and the outcome), with a true causal parameter equal to 2. The remaining results are provided in the Supplementary Materials. The boxplot of causal parameter estimates is presented in Fig. 1 (Supplementary Figs. S1 and S8). GOALDeR produced nearly unbiased estimates across all sample sizes, and the precision of the estimates was enhanced as *n* increased. In Scenario 2, when either the GPS model or outcome model was nonlinear (CoMt and MoCt), GOALDeR could still yield nearly unbiased estimates across all sample sizes (Fig. 2 and Supplementary Fig. S12) despite having unsatisfactory performance in variable selection (Supplementary Figs. S5 to S7 and S13 to S15). Under the setting where the outcome model is nonlinear and the GPS model is linear (MoCt), the variability of the estimates became smaller as the sample size increased. Not surprisingly, the estimates became biased when both the GPS and outcome models were nonlinear (MoMt; Fig. 2 and Supplementary Fig. S12).

**Fig. 3:**
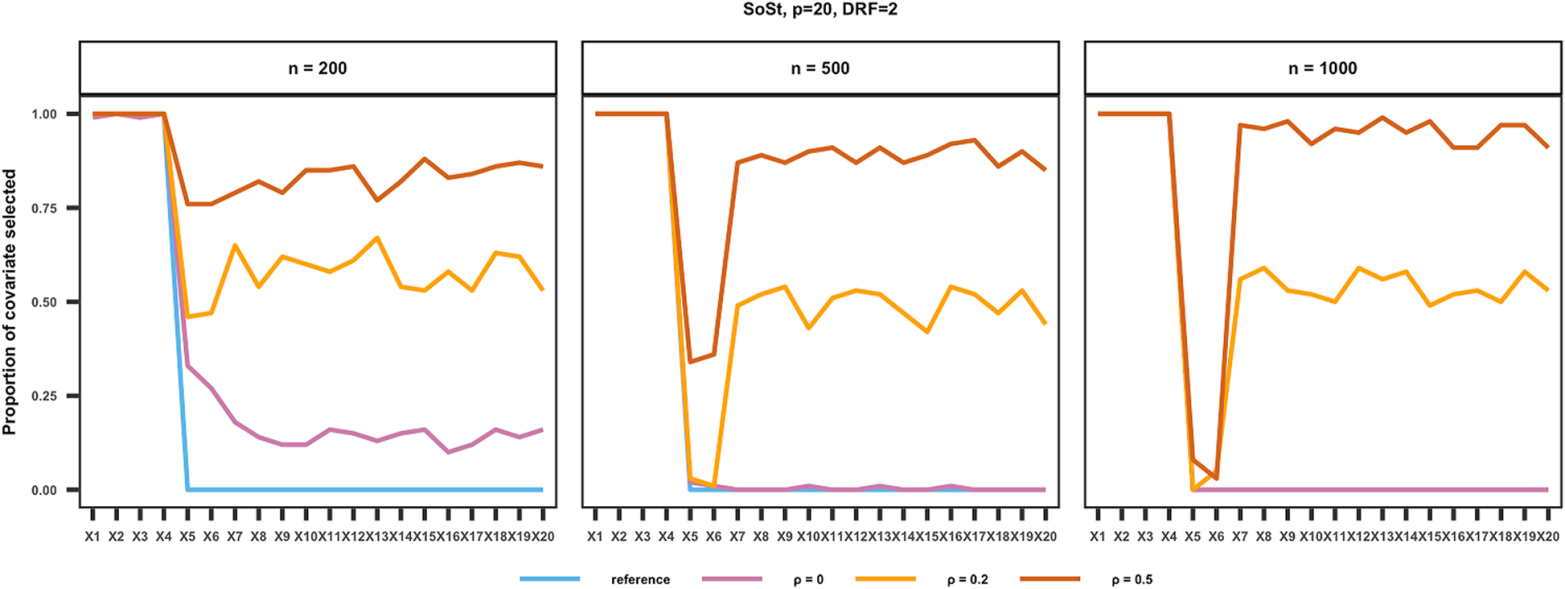
Illustrations with a modest *p* = 20. The probability of covariate selection being balanced over 100 simulations under the setting where the confounders were strongly correlated with both the treatment and the outcome (SoSt) and η = 2

The percentage of each covariate being selected under Scenario 1 is shown in Fig. 3 (Supplementary Figs. S2 to S4 and S9 to S11). We present the results for SoSt, with a true causal parameter equal to 2. The remaining results are presented in the Supplementary Materials. In general, the likelihood of selecting IVs decreased sharply with *n* increased. To illustrate no correlation between covariates, the average proportion of selecting IVs was 30% when *n* = 200, decreasing to 1.5% when *n* = 500, and further decreasing to 0 when *n* = 1000. The selection of IVs and spurious covariates increased as the correlation between covariates increased. Although GOALDeR may underselect confounders that are weakly correlated with outcome (Supplementary Figs. S4 and S11), it still yielded nearly unbiased estimates (Supplementary Figs. S1 and S8). Additionally, the GOALDeR showed a similar variable selection pattern when there was a large number of covariates.

### Estimation and testing under scenario 1 with a large number of covariates

**Fig. 4:**
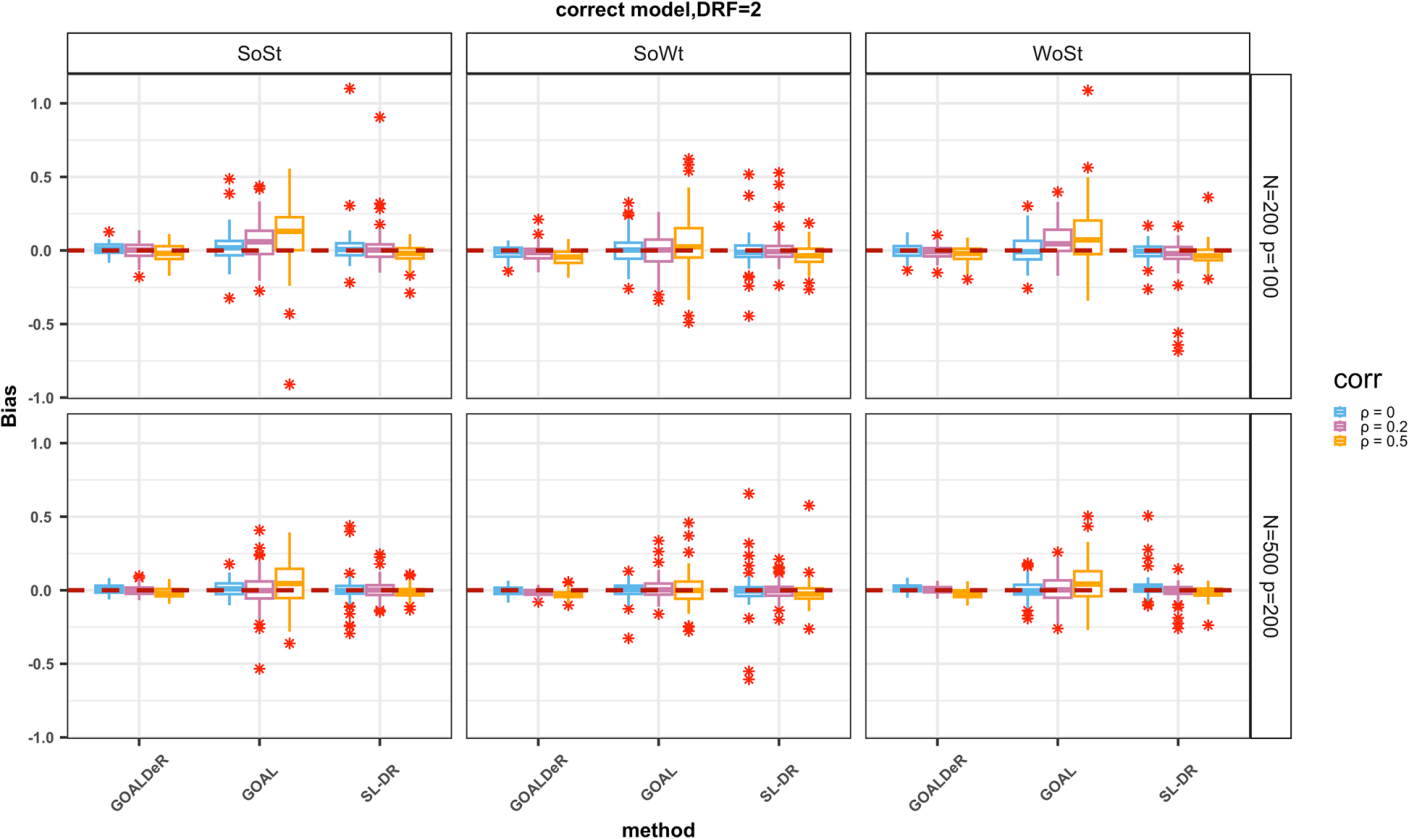
Boxplot of the bias for causal parameters in the dose–response function (DRF) by our method and GOAL, SL-DR under Scenario 1 with η = 2. The zero reference line is indicated by a dotted line, and the asterisks represent outliers

In Scenario 1, we compared the accuracy and precision of causal estimates between GOALDeR, GOAL, and SL-DR under varying strengths between confounders and both the treatment and outcome. The bias of parameter estimates in the DRF was used to evaluate accuracy. The bias distribution is shown in Fig. 4, and the summary statistics for Scenario 1 are listed in Table 2. GOALDeR showed nearly unbiased estimates across all three settings (Fig. 4; Table 2). The root mean squared error (RMSE) and the empirical standard error of the estimates were used to assess precision. The precision of GOALDeR was slightly enhanced when *n* = 500 compared to when *n* = 200. Interestingly, the accuracy and precision of GOALDeR were slightly impacted by the correlations between covariates (Fig. 4; Table 2). In contrast, as previously observed, the bias and variability (RMSE and empirical standard error) of GOAL increased as the correlations between covariates increased and the *n*/*p* ratio decreased. Compared with GOALDeR, SL-DR provided similar estimation accuracy, but the precision was significantly worse than that of GOALDeR (Fig. 4; Table 2). The reason is presumably owing to ignoring the negative effects of IVs when fitting the GPS model.

**Table 2:** Summary statistics of the performance under scenario 1 with the true parameter of DRF = 2

		*N* = 500, *P* = 200									*N* = 200, *P* = 100								
		DRF	Est_	Est_	Est_	Boot_	Boot_	Boot_	emp_	RMSE	DRF	Est_	Est_	Est_	Boot_	Boot_	Boot_	emp_	RMSE
			Std	Coverage	Power	std	coverage	power	std			Std	Coverage	Power	std	coverage	power	std	
SoSt rho = 0	GOALDeR	2.009	0.025	0.85	1	0.041	0.98	1	0.033	0.034	2.01	0.034	0.9	1	0.061	1	1	0.038	0.04
	SL-DR	2.002	0.037	0.79	1	-	-	-	0.087	0.087	2.021	0.039	0.63	1	-	-	-	0.128	0.129
	GOAL	2.012	0.1	1	1	-	-	-	0.052	0.053	2.025	0.162	1	1	-	-	-	0.106	0.109
SoWt rho = 0	GOALDeR	1.995	0.027	0.9	1	0.048	1	1	0.031	0.031	1.986	0.04	0.87	1	0.065	0.98	1	0.047	0.048
	SL-DR	1.997	0.047	0.8	1	-	-	-	0.125	0.124	1.994	0.049	0.79	1	-	-	-	0.102	0.102
	GOAL	1.998	0.087	0.99	1	-	-	-	0.056	0.056	2.004	0.151	1	1	-	-	-	0.096	0.096
WoSt rho = 0	GOALDeR	2.012	0.022	0.87	1	0.041	1	1	0.028	0.031	1.998	0.033	0.81	1	0.061	0.98	1	0.051	0.051
	SL-DR	2.02	0.033	0.78	1	-	-	-	0.073	0.075	1.995	0.032	0.64	1	-	-	-	0.06	0.06
	GOAL	2.002	0.087	0.96	1	-	-	-	0.064	0.063	2.002	0.133	0.97	1	-	-	-	0.095	0.095
SoSt rho = 0.2	GOALDeR	2.002	0.021	0.83	1	0.04	0.98	1	0.032	0.032	2.001	0.031	0.75	1	0.061	0.97	1	0.056	0.055
	SL-DR	2.004	0.033	0.57	1	-	-	-	0.063	0.063	2.017	0.043	0.66	1	-	-	-	0.119	0.12
	GOAL	2.003	0.148	0.99	1	-	-	-	0.117	0.116	2.066	0.185	0.97	1	-	-	-	0.132	0.147
SoWt rho = 0.2	GOALDeR	1.985	0.025	0.9	1	0.046	1	1	0.027	0.031	1.982	0.038	0.78	1	0.066	0.98	1	0.052	0.055
	SL-DR	1.999	0.04	0.81	1	-	-	-	0.061	0.061	2.004	0.047	0.7	1	-	-	-	0.098	0.097
	GOAL	2.01	0.13	0.99	1	-	-	-	0.072	0.073	1.998	0.2	1	1	-	-	-	0.117	0.116
WoSt rho = 0.2	GOALDeR	2.003	0.021	0.9	1	0.038	1	1	0.025	0.025	1.989	0.031	0.81	1	0.062	0.98	1	0.044	0.045
	SL-DR	1.992	0.033	0.8	1	-	-	-	0.055	0.055	1.968	0.04	0.61	1	-	-	-	0.123	0.126
	GOAL	2.01	0.122	0.99	1	-	-	-	0.098	0.098	2.068	0.159	0.95	1	-	-	-	0.116	0.134
SoSt rho = 0.5	GOALDeR	1.983	0.024	0.81	1	0.041	0.96	1	0.034	0.038	1.984	0.031	0.67	1	0.066	0.98	1	0.059	0.061
	SL-DR	1.986	0.023	0.62	1	-	-	-	0.039	0.041	1.98	0.027	0.52	1	-	-	-	0.062	0.065
	GOAL	2.046	0.183	0.99	1	-	-	-	0.146	0.152	2.112	0.223	0.9	0.99	-	-	-	0.213	0.24
SoWt rho = 0.5	GOALDeR	1.973	0.029	0.85	1	0.047	0.98	1	0.029	0.04	1.955	0.039	0.68	1	0.071	0.95	1	0.056	0.072
	SL-DR	1.983	0.034	0.64	1	-	-	-	0.08	0.082	1.963	0.037	0.59	1	-	-	-	0.07	0.079
	GOAL	2.005	0.186	0.99	1	-	-	-	0.114	0.114	2.005	0.186	0.99	1	-	-	-	0.114	0.114
WoSt rho = 0.5	GOALDeR	1.979	0.024	0.74	1	0.04	0.96	1	0.034	0.04	1.977	0.032	0.74	1	0.065	0.97	1	0.056	0.06
	SL-DR	1.987	0.025	0.77	1	-	-	-	0.039	0.041	1.971	0.026	0.48	1	-	-	-	0.065	0.07
	GOAL	2.045	0.16	0.98	1	-	-	-	0.136	0.143	2.045	0.16	0.98	1	-	-	-	0.136	0.143

Notations: DRF = 2, the parameter of DRF is 2; rho = 0, rho = 0.2, and rho = 0.5, there are no, moderate and strong correlations between covariates; emp_std: the empirical standard deviation of the estimates; Est_Std: the mean of standard deviation estimates using sandwich-type estimator (GOAL) or regression of the treatment on pseudo-outcome (GOALDeR and SL-DR); Boot_Std: the mean of bootstrapped standard deviation; coverage: coverage probability of the 95% confidence interval; RMSE: root mean square error calculated as $\:\text{R}\text{M}\text{S}\text{E}=\sqrt{\frac{1}{B}{\sum\:}_{b=1}^{B}{\left({\widehat{\eta\:}}_{b}-\eta\:\right)}^{2}}$

The standard deviation (SD) was estimated using the regression of the treatment on pseudo-outcome for the GOALDeR and SL-DR methods, and the sandwich-type variance estimator for the GOAL method [25]. The coverage probability of the 95% confidence interval (CI) and power were used to assess statistical testing. As shown in Table 2, for GOALDeR, the estimated SDs were lower than the empirical standard errors in most cases, resulting in the coverage of the 95% CI being less than 95% (ranging from 67 to 90%). For SL-DR, the estimated SDs were significantly lower than the empirical standard errors, and the coverage of the 95% CI (ranging from 48 to 81%) was consistently lower than that of the GOALDeR method. This implied that the estimated SDs for GOALDeR and SL-DR methods were underestimated. For GOAL, the estimated SDs were larger than the empirical standard errors, resulting in the coverage of the 95% CI tending to be conservative (ranging from 95 to 100%). We also estimated the SD using the bootstrap method for the GOALDeR method and found that the bootstrap SD was slightly higher than the empirical standard errors, and the corresponding coverage was around 95% in most cases.

GOALDeR, GOAL, and SL-DR had similar power, which was 1 in all three settings (Table 2). Furthermore, we also explored the performance of each method when the DRF parameter was set to 0.4 and 0.7 to investigate the impact of effect size on statistical testing. The results for $\:\eta\:=0.4\:\text{a}\text{n}\text{d}\:\:0.7$ are presented in Supplementary Tables S3 and S4, which were similar to those for $\:\eta\:=2$ except for the power. When the DRF parameter decreased from 2 to 0.4, the power for GOALDeR remained consistently at 1, while it slightly decreased for SL-DR and significantly decreased for the GOAL method.

### Estimation and testing under scenario 2 with a large number of covariates

**Fig. 5:**
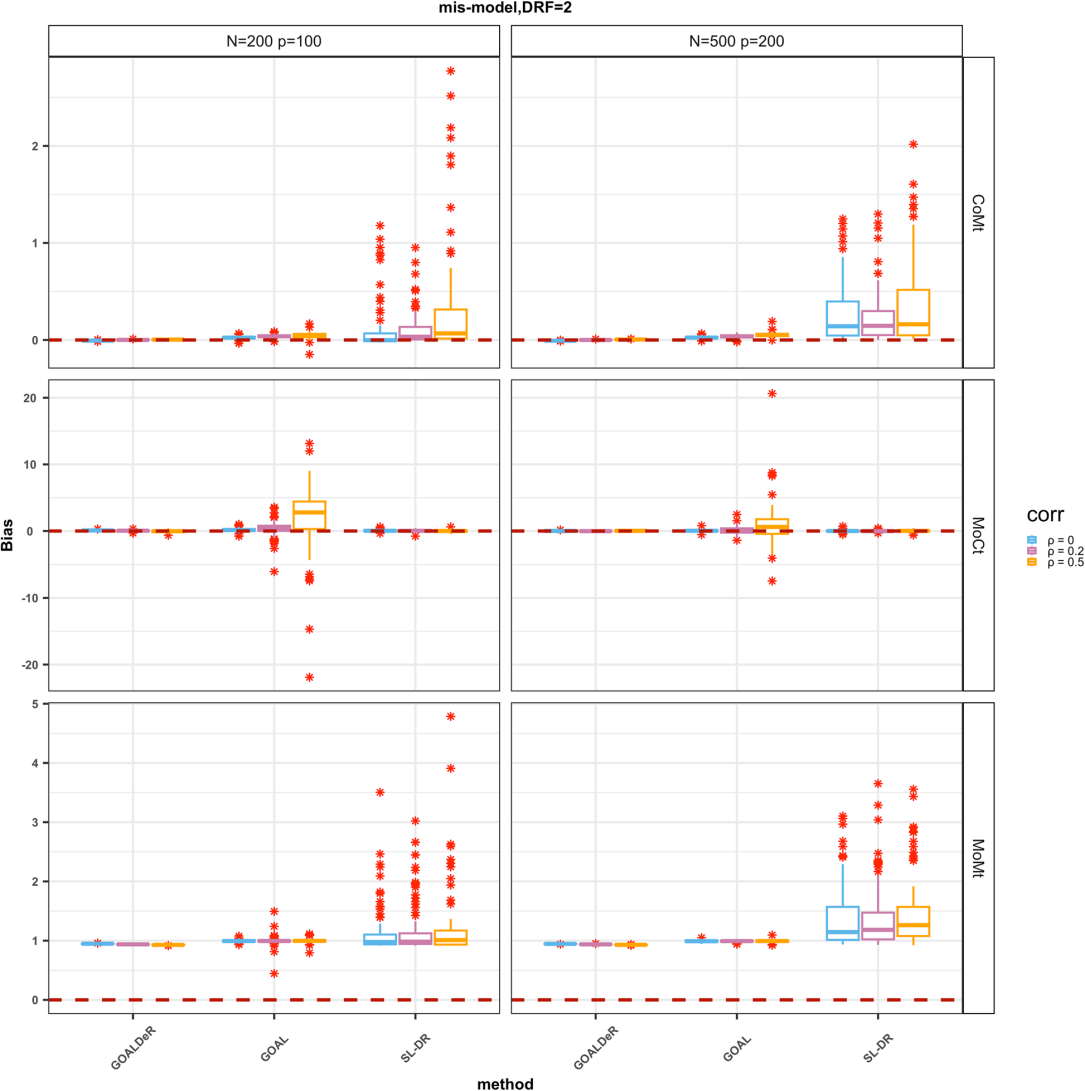
Boxplot of the bias for the causal parameters in the dose–response function (DRF) using our method and GOAL, and SL-DR under Scenario 2 with η = 2. The zero reference line is indicated by a dotted line, and the asterisks represent outliers

In Scenario 2, we assessed the double robustness of GOALDeR, GOAL, and SL-DR. The bias distribution of parameter estimates in DRF is shown in Fig. 5, and the summary statistics for Scenario 2 are listed in Table 3. GOALDeR yielded estimates that were close to the true value 2 as long as one of the outcome and the GPS models were correctly specified, and the biases were less impacted by the correlation between covariates and the *n/p* ratio. This indicated the double robustness of GOALDeR (Table 3; Fig. 5). In the setting of MoCt, the variability (RMSE and empirical standard error) of the estimates by GOALDeR became large as the correlation between covariates increased, especially when the *n*/*p* ratio was small (*n*/*p* = 200/100). The SL-DR method also tended to be doubly robust. Compared to GOALDeR, SL-DR provided estimates with smaller biases and slightly higher RMSE in the MoCt setting. However, when only the GPS model was non-linear (CoMt), its biases and RMSE were significantly larger than those of the GOALDeR method, especially when there was a strong correlation among covariates. In contrast, GOAL became biased when the outcome model was incorrectly specified because it relies on the assumption that the outcome model is linear for variable selection. In the MoMt setting, all three approaches were biased, with SL-DR exhibiting the largest biases and RMSE.

**Table 3:** Summary statistics of the performance under scenario 2 with the true parameter of DRF = 2

		*N* = 500, *P* = 200									*N* = 200, *P* = 100								
		DRF	Est_	Est_	Est_	Boot_	Boot_	Boot_	emp_	RMSE	DRF	Est_	Est_	Est_	Boot_	Boot_	Boot_	emp_	RMSE
			Std	Coverage	Power	std	coverage	power	std			Std	Coverage	Power	std	coverage	power	std	
CoMt rho = 0	GOALDeR	1.993	0.002	0.12	1	0.006	0.93	1	0.002	0.007	1.993	0.004	0.58	1	0.0079	0.97	1	0.003	0.007
	SL-DR	2.257	0.054	0.08	1	-	-	-	0.297	0.392	2.094	0.032	0.14	1	-	-		0.242	0.259
	GOAL	2.024	0.012	0.34	1	-	-	-	0.014	0.028	2.023	0.014	0.48	1	-	-		0.016	0.028
MoCt rho = 0	GOALDeR	2.022	0.055	0.96	1	0.079	0.99	1	0.049	0.054	2.078	0.078	0.83	1	0.116	0.97	1	0.083	0.114
	SL-DR	2.007	0.077	0.77	1	-	-	-	0.138	0.137	2.039	0.074	0.61	1	-	-		0.149	0.153
	GOAL	2.04	2.292	1	0	-	-	-	0.177	0.181	2.165	3.067	1	0	-	-		0.298	0.339
MoMt rho = 0	GOALDeR	2.946	0.003	0	1	0.009	0	1	0.003	0.946	2.948	0.004	0	1	0.012	0	1	0.004	0.948
	SL-DR	3.388	0.09	0	1	-	-	-	0.546	1.49	3.129	0.058	0	1	-	-		0.391	1.194
	GOAL	2.992	0.019	0	1	-	-	-	0.02	0.992	2.994	0.019	0	1	-	-		0.022	0.994
CoMt rho = 0.2	GOALDeR	2	0.002	0.73	1	0.0066	1	1	0.003	0.003	2.001	0.004	0.98	1	0.0096	1	1	0.003	0.003
	SL-DR	2.227	0.044	0.01	1	-	-	-	0.261	0.345	2.109	0.031	0.12	1	-	-	-	0.171	0.202
	GOAL	2.037	0.016	0.28	1	-	-	-	0.017	0.041	2.04	0.015	0.18	1	-	-	-	0.015	0.042
MoCt rho = 0.2	GOALDeR	1.986	0.048	0.79	1	0.099	0.99	1	0.072	0.073	2.027	0.059	0.65	1	0.1508	0.98	1	0.13	0.132
	SL-DR	1.996	0.054	0.67	1	-	-	-	0.105	0.104	2.03	0.051	0.46	1	-	-	-	0.16	0.162
	GOAL	2.074	2.673	1	0.01	-	-	-	0.598	0.599	2.329	3.717	1	0.01	-	-	-	1.182	1.221
MoMt rho = 0.2	GOALDeR	2.937	0.003	0	1	0.009	0	1	0.004	0.937	2.938	0.004	0	1	0.013	0	1	0.004	0.938
	SL-DR	3.387	0.086	0	1	-	-	-	0.542	1.488	3.182	0.065	0	1	-	-	-	0.452	1.264
	GOAL	2.99	0.014	0	1	-	-	-	0.016	0.99	2.997	0.03	0.01	1	-	-	-	0.084	1
CoMt rho = 0.5	GOALDeR	2.005	0.003	0.53	1	0.0084	1	1	0.002	0.005	2.004	0.004	0.8	1	0.012	1	1	0.004	0.006
	SL-DR	2.352	0.062	0	1	-	-	-	0.434	0.557	2.31	0.074	0	1	-	-	-	0.561	0.638
	GOAL	2.052	0.016	0.14	1	-	-	-	0.025	0.058	2.046	0.024	0.31	1	-	-	-	0.033	0.057
MoCt rho = 0.5	GOALDeR	2.023	0.041	0.52	1	0.142	0.99	1	0.11	0.111	1.963	0.058	0.41	1	0.209	0.96	1	0.189	0.191
	SL-DR	2.014	0.04	0.37	1	-	-	-	0.132	0.132	2.009	0.044	0.32	1	-	-	-	0.183	0.182
	GOAL	2.943	4.044	1	0.04	-	-	-	3.04	3.169	4.116	5.118	0.84	0.26	-	-	-	4.74	5.169
MoMt rho = 0.5	GOALDeR	2.929	0.003	0	1	0.0104	0	1	0.003	0.929	2.928	0.005	0	1	0.016	0	1	0.004	0.928
	SL-DR	3.455	0.098	0	1	-	-	-	0.582	1.566	3.197	0.075	0	1	-	-	-	0.582	1.33
	GOAL	2.991	0.016	0	1	-	-	-	0.022	0.992	2.995	0.022	0	1	-	-	-	0.035	0.996

Notations: DRF = 2, the parameter of DRF is 2; rho = 0, rho = 0.2, and rho = 0.5, there are no, moderate and strong correlations between covariates; emp_std: the empirical standard deviation of the estimates; Est_Std: the mean of standard deviation estimates using sandwich-type estimator (GOAL) or regression of the treatment on pseudo-outcome (GOALDeR and SL-DR); Boot_Std: the mean of bootstrapped standard deviation; coverage: coverage probability of the 95% confidence interval; RMSE: root mean square error calculated as $\:\text{R}\text{M}\text{S}\text{E}=\sqrt{\frac{1}{B}{\sum\:}_{b=1}^{B}{\left({\widehat{\eta\:}}_{b}-\eta\:\right)}^{2}}$

As shown in Table 3, when one of the models was correctly specified (CoMt and MoCt), the estimated SDs of GOALDeR were less than or equal to the empirical standard errors, resulting in the coverage being less than 95% in most cases. The bootstrap SDs were higher than the empirical standard errors, and the corresponding coverage tended to be conservative (ranging from 93 to 100%). For the SL-DR method, the estimated SDs were significantly less than the empirical standard errors, and the coverage of the 95% CI (ranging from 0 to 77%) was consistently lower than that of the GOALDeR method. For the GOAL method, the estimated SDs were nearly equal to the empirical standard errors in the setting of CoMt, and the corresponding coverage was less than 95%. In the setting of MoCt, the estimated SDs of GOAL were significantly larger than the empirical standard errors, and the coverage was conservative. In the setting of MoMt, the coverage of all three methods was 0 because of the large bias.

The power of GOALDeR and SL-DR was always 1 in all three settings (Table 3). In contrast, the power of GOAL was significantly reduced when only the outcome model was incorrectly specified (MoCt). When the DRF parameter decreased from 2 to 0.4, the power for GOALDeR remained consistently at 1, while it slightly decreased for SL-DR. The coverage for the GOALDeR and SL-DR methods decreased.

## Real data applications

We applied GOALDeR and SL-DR to study causal relationships between epigenetic age acceleration and AD. The results of the GOAL method have been reported in our previous study [25]. We followed steps similar to those implemented in the GOAL method to collect datasets, calculate DNA methylation (DNAm) age, and process and identify potential confounders [25]. Briefly, we downloaded seven datasets from the Gene Expression Omnibus database according to the inclusion and exclusion criteria. The accession numbers are GSE105109 [37], GSE125895 [38], GSE134379 [39], GSE59685 [40], GSE66351 [41], GSE80970 [42], and GSE109627 [43], covering four brain regions: frontal cortex (FC), temporal cortex (TC), entorhinal cortex (ERC), and cerebellum (CRB). The ‘cortical DNAm clock’ was used to estimate DNAm age, which is a measure of biological age [44]. The residuals of the regression model of chronological age on DNAm age were defined as epigenetic age acceleration. We considered chronological age and gender to be recognized risk factors for AD, and the datasets with raw data also controlled for the proportion of neuronal cells. Additionally, we regarded whole-genome CpG sites as potential covariates, as they may contain confounders and prognostic covariates or act as surrogates for these two types of covariates. Initially, we selected potential adjustment CpG sites through epigenome-wide association study (EWAS) meta-analysis for each brain region, keeping the top *K* CpG sites with the smallest Bonferroni-adjusted *P* values. The value of *K* for each brain region was determined as follows: *K* = minimum sample size in the specific brain region − (number of known covariates + 2), since GOALDeR is not directly applicable when *p* > *n*.

Table 4 shows the estimated causal DRF of the GOALDeR and SL-DR methods between epigenetic age acceleration and AD across four brain regions. For the GOALDeR method, the four brain regions showed consistent results that there was no statistically significant dose–response relationship between epigenetic age acceleration and AD (*P* > 0.05). For the SL-DR method, the results for the four regions were inconsistent. We therefore performed a meta-analysis with a random-effects model (Supplementary Fig. S18) because there was heterogeneity among datasets (TC: $\:{I}^{2}=96.4\%,\:Q=111.76,\:P<\:0.0001$; FC: $\:{I}^{2}=$94.5%$\:,\:Q=54.63,\:P<\:0.0001$; ERC: $\:{I}^{2}=$92.8%$\:,\:Q=27.64,\:P<\:0.0001$; CRB: $\:{I}^{2}=$90.3%$\:,\:Q=30.95,\:P<0.0001$). The pooled odds ratios were 0.9985 (95% confidence interval: 0.9943–1.0027, *P* = 0.4883), 1.0006 (95% confidence interval: 0.9945–1.0067, *P* = 0.8550), 0.9643 (95% confidence interval: 0.8860–1.0496, *P* = 0.4008), and 0.9885 (95% confidence interval: 0.9510–1.0276, *P* = 0.5601), respectively.

**Table 4:** Summary statistics for the datasets and results of GOALDeR and SL-DR analyses across the four brain regions

Brain region	dataset	N (AD/Control)	Potential confounders	Results for GOALDeR				Results for SL-DR			
				DRF	sd(DRF)	t	*P*	DRF	sd(DRF)	t	*P*
**TC**	**GSE59685**	861 (489/ 372)	age, gender, 60 CPG sites	0.0021	0.0054	0.3997	0.6904	−0.0011	0.0016	−0.7056	0.4824
	**GSE66351**		age, gender, neuronal cell proportion, 60 CPG sites	−0.011	0.0089	−1.2425	0.2186	−0.007	0.0002	−30.746	***< 0.0001***
	**GSE80970**		age, gender, 60 CPG sites	0.0032	0.0037	0.8758	0.3826	0.0042	0.0016	2.5909	***0.0106***
	**GSE109627**		age, gender, 59 CPG sites	0.0004	0.0033	0.1092	0.9132	−0.0001	0.0009	−0.1107	0.912
	**GSE134379**		age, gender, neuronal cell proportion, 60 CPG sites	0.0097	0.0056	1.7423	0.0822	−0.0044	0.0051	−0.8533	0.394
**FC**	**GSE59685**	357 (192/165)	age, gender, 58 CPG sites	0.0068	0.0042	1.6141	0.1103	−0.0042	0.0022	−1.8791	0.0638
	**GSE66351**		age, gender, neuronal cell proportion, 58 CPG sites	−0.0027	0.0052	−0.5117	0.6107	−0.0031	0.0014	−2.2978	***0.025***
	**GSE80970**		age, gender, 58 CPG sites	−0.0026	0.004	−0.6533	0.5146	−0.0002	0.0016	−0.1314	0.8956
	**GSE125895**		age, gender, neuronal cell proportion, 57 CPG sites	0.0034	0.003	1.1252	0.2646	0.0094	0.0013	7.0841	***< 0.0001***
**ERC**	**GSE59685**	336 (212/124)	age, gender, 64 CPG sites	−0.0084	0.0051	−1.6233	0.1086	−0.129	0.0254	−5.0823	***< 0.0001***
	**GSE105109**		age, gender, neuronal cell proportion, 62 CPG sites	−0.0009	0.0027	−0.3418	0.7329	0.0106	0.0079	1.3435	0.1808
	**GSE125895**		age, gender, neuronal cell proportion, 64 CPG sites	−0.0002	0.0023	−0.0991	0.9214	0.0001	0.001	0.1012	0.9197
**CRB**	**GSE59685**	746 (445/301)	age, gender, 59 CPG sites	−0.0117	0.0069	−1.7066	0.0917	−0.0774	0.0164	−4.7099	***< 0.0001***
	**GSE105109**		age, gender, neuronal cell proportion, 46 CPG sites	0.0022	0.0108	0.2058	0.8371	−0.0001	0.0062	−0.022	0.9824
	**GSE125895**		age, gender, neuronal cell proportion, 58 CPG sites	0.0011	0.0071	0.1581	0.8749	0.0087	0.0032	2.6944	***0.009***
	**GSE134379**		age, gender, neuronal cell proportion, 44 CPG sites	0.0118	0.0078	1.52	0.1293	0.0133	0.0035	3.7385	***0.0002***

Notations: DRF, the causal parameter of the DRF; N, sample size

In summary, the GOALDeR and SL-DR analyses found that there was no statistically significant dose-response association between epigenetic age acceleration and AD, which is consistent with the results of the GOAL method [25]. In addition, the results of the SL-DR method showed greater variability than those of GOALDeR, which is consistent with our simulation results.

## Discussion

We developed a new approach, GOALDeR, to estimate the linear or nonlinear DRF in high dimensions. Our extensive simulation studies, conducted under both correct and incorrect model specifications, indicated that GOALDeR can produce nearly unbiased estimates as long as either the outcome or GPS model is correctly specified. Therefore, it shows doubly robust empirically. The performance of GOALDeR is less impacted by the *n*/*p* ratio and correlated covariates. GOALDeR can also achieve statistical power and 95%CI coverage that are comparable to those of other methods.

Our simulations show that GOAL requires a linear outcome model to produce unbiased estimates, but the accuracy and precision worsen when there are correlated covariates or the *n*/*p* ratio is small. These results are consistent with those of previous studies [25]. The SL-DR requires the user to specify the conditional and marginal distributions of the treatment [22–24]. In our simulations, we assumed normal distributions for the treatment and the GPS both in the data-generating process and in the estimation of balance weights for SL-DR. This setting may partly contribute to the nearly doubly robust performance of SL-DR and explain why SL-DR performs less accurately and precisely than GOALDeR when the GPS model is misspecified and the outcome model is correctly specified. Additionally, the variability of estimates for SL-DR is greater than that of GOALDeR. This may be because SL-DR ignores the influence of IVs when estimating the GPS [16–21].

The outstanding performance of GOALDeR in estimation accuracy and precision may be attributed to the following: (1) GOALDeR uses a balance-based method to estimate balance weights, thereby avoiding the need to specify distributions for the treatment and GPS [15]; (2) GOALDeR uses a distance correlation coefficient as the measure to assess covariate balance, thereby avoiding the need to specify the orders of moment of both the covariates and the treatment to decorrelate [15]; (3) GOALDeR constructs penalty weights based on conditional correlation between the outcome and covariates without depending on the outcome model, thereby achieving exclusion IVs and estimation DRF in the doubly robust framework [21]. However, as with most existing methods [16, 21, 22, 25, 29], GOALDeR lacks a standard deviation estimator to guarantee a valid confidence interval. Here, GOALDeR uses the regression coefficient of the treatment on pseudo-outcome to obtain an inference of DRF. The corresponding power consistently equaled 1, while the coverage of the 95% CI was often less than the nominal value, suggesting that the SDs were underestimated. This underestimation may be due to the estimated SD failing to adequately capture the variability of variable selection. We also employed the bootstrap method to estimate the SD and found that (i) the bootstrap SDs were slightly higher than the empirical standard errors when both the GPS and outcome models were correctly specified, resulting in coverage probabilities around the nominal value in most cases; (ii) the bootstrap SDs were moderately higher than the empirical standard errors when either the GPS or the outcome model was correctly specified, leading to coverage probabilities that tended to be conservative (greater than the nominal value) in most cases. Although the bootstrap method tends to improve the statistical tests, it does not completely resolve the inference problem after variable selection [45]. Therefore, further research on the development of a valid and widely applicable variance estimator after variable selection is a possible topic in future work [46].

In summary, this study proposed a doubly robust estimator for continuous treatment and high-dimensional covariates. Within the framework of the doubly robust (DR) estimator, the proposed GOALDeR method combined a variable selection technique for causal inference to ensure unbiased and statistically efficient estimation, along with a balance-based method that was robust to misspecification of the distributions required for GPS methods. Simulation results and real data analyses provided empirical evidence that GOALDeR achieved double robustness, offering improved accuracy and precision compared to existing methods. We also provided an R package for implementing the GOALDeR method, available at https://github.com/QianGao-SXMU/GOALDeR.

## Supplementary Information

Supplementary Material 1

## Data Availability

No datasets were generated or analysed during the current study.
