# Supplementary material for: A doubly robust estimator for continuous treatments in high dimensions
Source: BMC Med Res Methodol. 2025 Feb 13;25:35. doi: 10.1186/s12874-025-02488-3 (PMC11823051; doi:10.1186/s12874-025-02488-3)
Supplement: Supplementary file 1 — Supplementary Material 1 [file 12874_2025_2488_MOESM1_ESM.docx]

Supplemental file for “A doubly robust estimator for continuous treatments in high dimensions”

Qian Gao^1^, Jiale Wang^1^, Ruiling Fang^1^, Hongwei Sun^2^, Tong Wang^1*^

^1^ Department of Health Statistics, School of Public Health, MOE Key Laboratory of Coal Environmental Pathogenicity and Prevention, Shanxi Medical University, Taiyuan, China

^2^ Department of Health Statistics, School of Public Health and Management, Binzhou Medical University, Yantai, China

**Correspondence to:* Tong Wang, Department of Health Statistics, School of Public Health, Shanxi Medical University, No.56 Xinjian South Road, 030001, Taiyuan, China; Tel: +86-351-4135397; Fax: +86-351-4135998, Email: [tongwang@sxmu.edu.cn](mailto:tongwang@sxmu.edu.cn).

# Abstract

**Background:** Generalized propensity score (GPS) methods have become popular for estimating causal relationships between a continuous treatment and an outcome in observational studies with rich covariate information. The presence of rich covariates enhances the plausibility of the unconfoundedness assumption. Nonetheless, it is also crucial to ensure the correct specification of both marginal and conditional treatment distributions, beyond the assumption of unconfoundedness.

**Method:** We address limitations in existing GPS methods by extending balance-based approaches to high dimensions and introducing the Generalized Outcome-Adaptive LASSO and Doubly Robust Estimate (GOALDeR). This novel approach integrates a balance-based method that is robust to the misspecification of distributions required for GPS methods, a doubly robust estimator that is robust to the misspecification of models, and a variable selection technique for causal inference that ensures an unbiased and statistically efficient estimation.

**Results:** Simulation studies showed that GOALDeR was able to generate nearly unbiased estimates when either the GPS model or the outcome model was correctly specified. Notably, GOALDeR demonstrated greater precision and accuracy compared to existing methods and was slightly affected by the covariate correlation structure and ratio of sample size to covariate dimension. Real data analysis revealed no statistically significant dose-response relationship between epigenetic age acceleration and Alzheimer's disease.

**Conclusion:** In this study, we proposed GOALDeR as an advanced GPS method for causal inference in high dimensions, and empirically demonstrated that GOALDeR is doubly robust, with improved accuracy and precision compared to existing methods. The R package is available at <https://github.com/QianGao-SXMU/GOALDeR>.

**Keywords:** causal inference, doubly robust, high-dimensional data, generalized propensity score

# The implementation of Generalized outcome-adaptive LASSO

The generalized outcome-adaptive LASSO (GOAL) [1] method consists of three steps:

(1) Step 1: **Variable selection based on outcome-adaptive LASSO**. The objective function can be written as:

$$\hat{\boldsymbol{\alpha}}=arg\min_{\boldsymbol{\alpha}} \left\| T-\sum_{j=1}^{p} Z_{j}\alpha_{j} \right\|^{2}+\lambda_{n}\sum_{j=1}^{p} \hat{w}_{j}\left| \alpha_{j} \right|$$

where $\hat{w}_{j}=\left| \tilde{\beta}_{j} \right|^{-\gamma}$, $\gamma>1$，$\left( \tilde{\boldsymbol{\beta}},\tilde{\eta} \right)=\arg\min_{\boldsymbol{\beta},\eta} \left\| Y-\eta T-\sum_{j=1}^{p} Z_{j}\beta_{j} \right\|^{2}$. $\tilde{\beta}_{j}$ refers to the coefficient corresponding to the j^th^ covariate in the unpenalized “full” outcome regression model. Given $\gamma>1$ and $\lambda_{n}$ that satisfy $\lambda_{n}/\sqrt{n}\to0$ and $\lambda_{n}n^{\gamma/2-1}\to\infty$ for consistency in variable selection, we can select a set of variables.

(2) Step 2: **Choosing** $\boldsymbol{\lambda}_{\boldsymbol{n}}$**.** The GOAL method selects the optimal $\lambda_{n}$ by minimizing dual-weight correlation (DWC).

$$DWC\left( \lambda_{n} \right)=\sum_{j=1}^{p} \left| \tilde{\beta}_{j} \right|\left| E\left( \hat{w}_{i}^{\lambda_{n}}T_{i}Z_{ij} \right) \right|$$

where $\hat{w}_{i}^{\lambda_{n}}$ is the weight for the i^th^ observation, and $\hat{w}_{i}^{\lambda_{n}}$ are estimated using the npCBGPS method with covariates selected according to Step 1 with $\lambda_{n}$; $\left| E\left( \hat{w}_{i}^{\lambda_{n}}T_{i}Z_{ij} \right) \right|$ is the weighted correlation coefficient between treatment and covariate j, reflecting the balance, and $\tilde{\beta}_{j}$ is the unpenalized estimate of coefficient for the j^th^ covariate in the “full” outcome model, reflecting the strength of association between covariates and outcome.

(3) Step 3: **Estimation based on inverse probability weighting (IPW)**. Given the optimal $\lambda_{n}$ and its corresponding balance weights, a marginal structural model approach is used to estimate the DRF. Under the four identification assumptions (Consistency, Positivity, Unconfoundedness, and Stable unit treatment assumption), a weighted linear or non-linear outcome regression model can be fitted to estimate DRF.

# Simulation results

## 1. Results under Scenarios 1 and 2 with a modest *p* = 20

## 1.1 Estimation under Scenarios 1 and 2 with a modest *p* = 20

In Scenario 1, both the GPS and outcome models were linear, and we established three settings by varying the strength of the relationship between confounders and both the outcome and treatment (SoSt, SoWt, and WoSt). The boxplot of causal parameter estimates for all three settings is shown in Fig. S1. As reported in the main manuscript, GOALDeR produced nearly unbiased estimates across all sample sizes, and the precision of the estimates improved as *n* increased.

In Scenario 2, we evaluated the double robustness of GOALDeR and established three settings where either the GPS or outcome model was incorrectly specified. In the settings of CoMt (where the outcome model was correctly specified and the GPS model was misspecified) and MoCt (where the outcome model was incorrectly specified and the GPS model was correctly specified), GOALDeR provided nearly unbiased estimates across all sample sizes (Fig. 2 in the main manuscript). These results indicate that GOALDeR is doubly robust. In the setting of MoMt, where neither the GPS model nor the outcome model was correctly specified, GOALDeR produced biased estimates (Fig. 2).


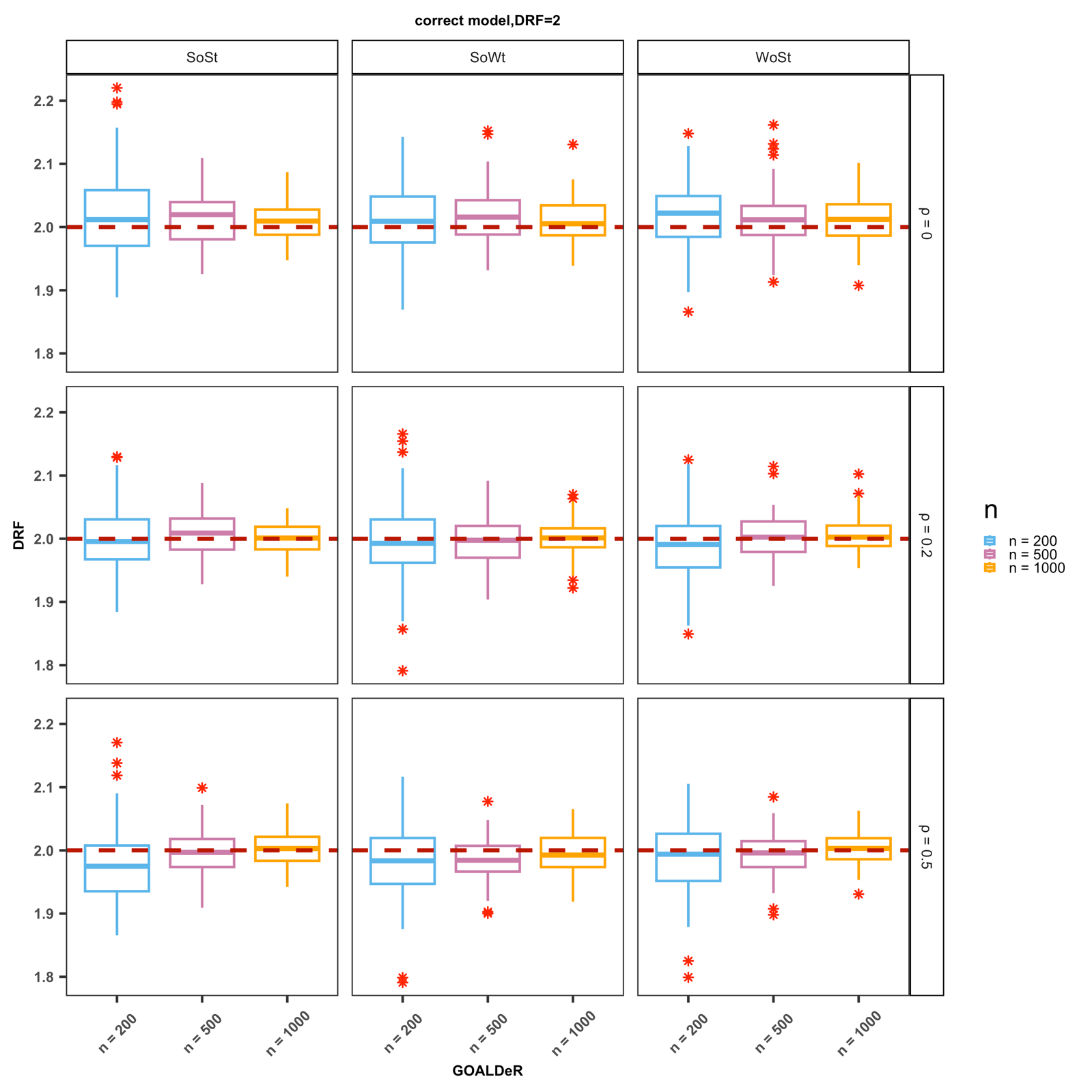


**Fig. S1.** Illustrations with a modest *p* = 20. Boxplot of parameters for the dose–response function (DRF) under Scenario 1 with η = 2. The true causal parameter of 2 is indicated by a dotted line, and the asterisks represent outliers.

## 1.2 Variable selection under Scenarios 1 and 2 with a modest *p*=20

In Figs. S2 to S4, we reported the percentage of each covariate being selected for all three settings under Scenario 1. The results for the SoSt setting with η = 2 were reported in the main manuscript; however, we include them here as well to facilitate comparisons across settings. As indicated in Figs. S2 to S4, GOALDeR selected nearly all confounders and prognostic covariates, and the selection of IVs decreased significantly as *n* increased. The likelihood of selecting IVs and spurious covariates increased as the correlation between covariates increased.


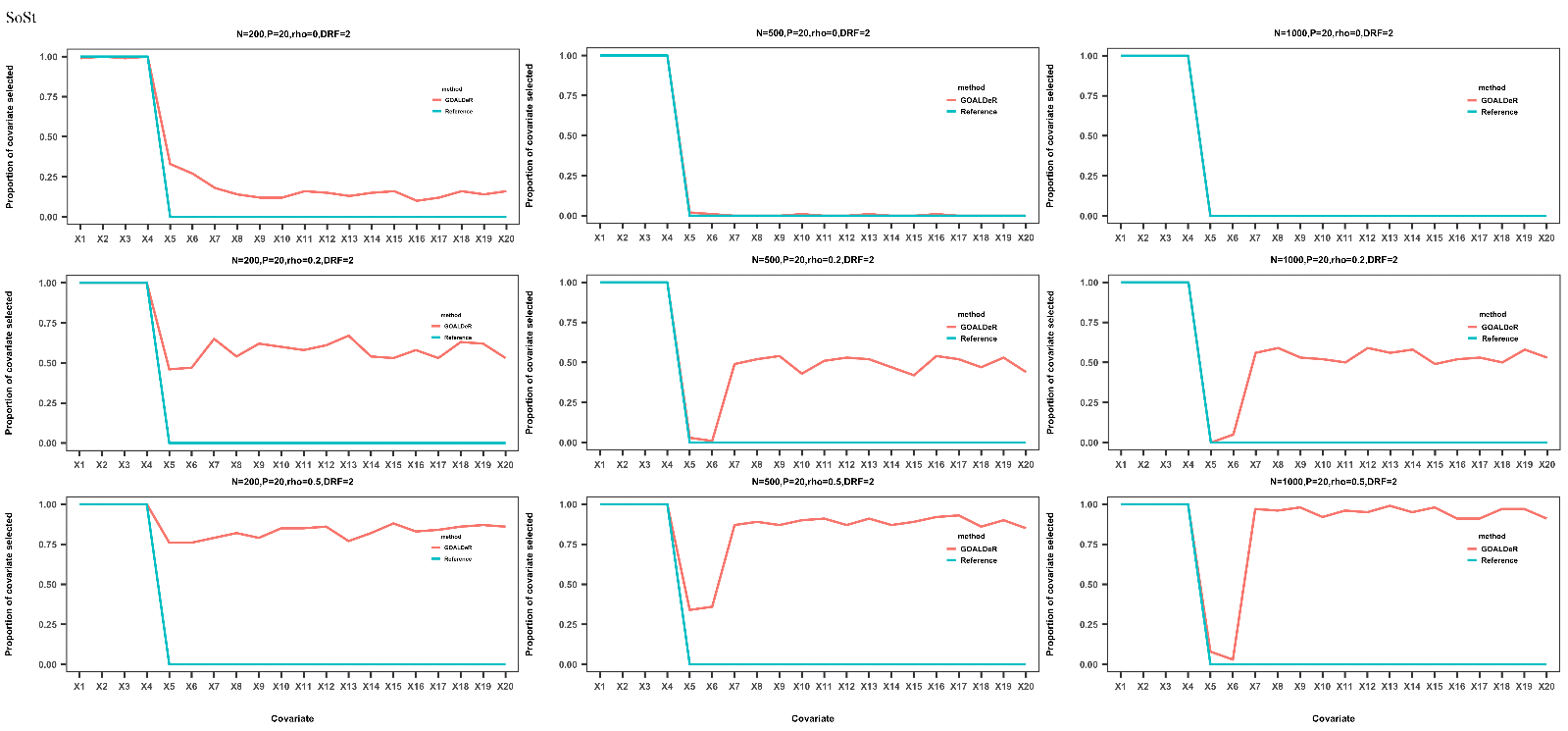


**Fig. S2.** Illustrations with a modest *p* = 20. The probability of covariate selection being balanced over 100 simulations under the setting where the confounders were strongly correlated with both the treatment and the outcome (SoSt) and $\eta=2$.


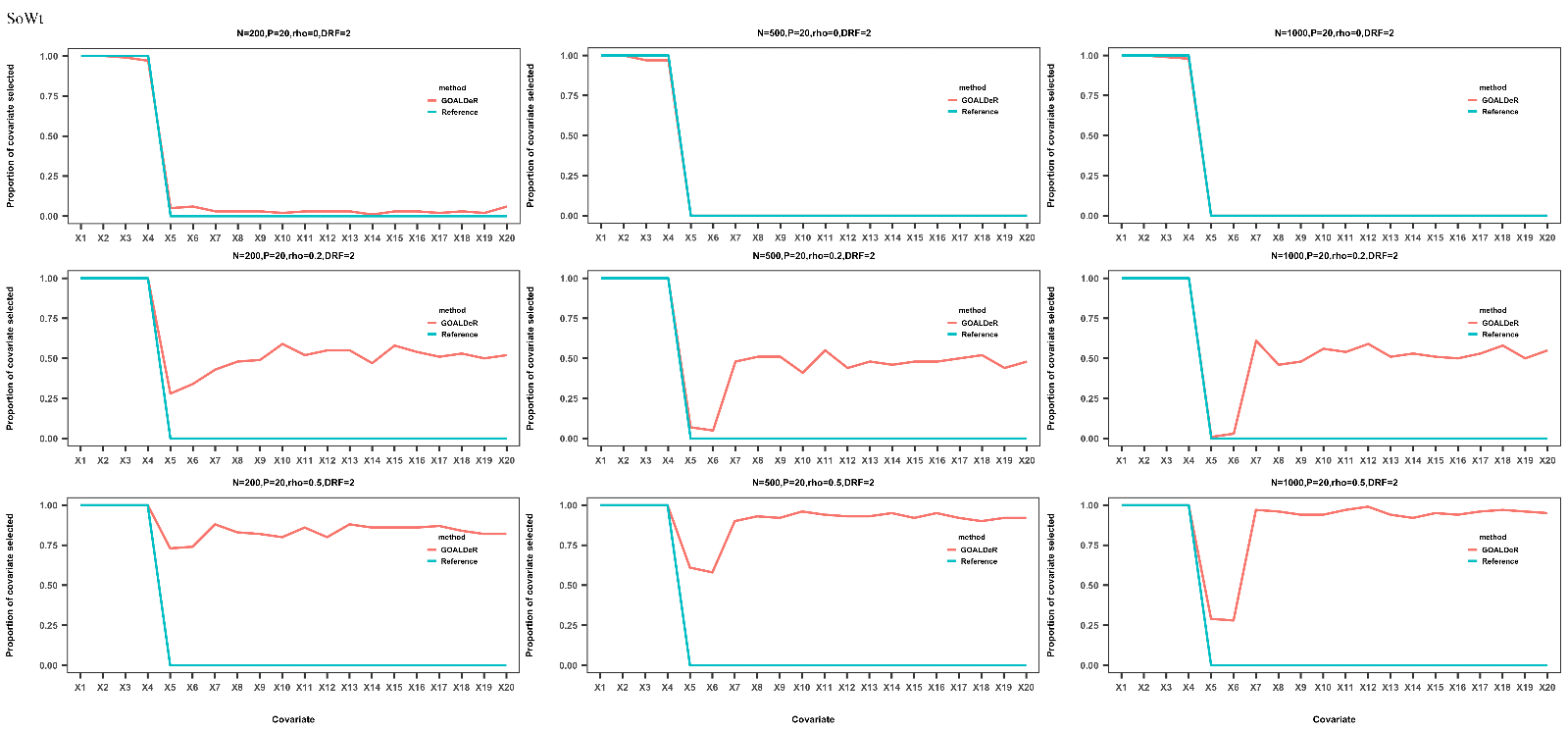


**Fig. S3.** Illustrations with a modest *p* = 20. The probability of covariate selection being balanced over 100 simulations under the setting where the confounders were relatively weakly correlated with the treatment (SoWt) and $\eta=2$.


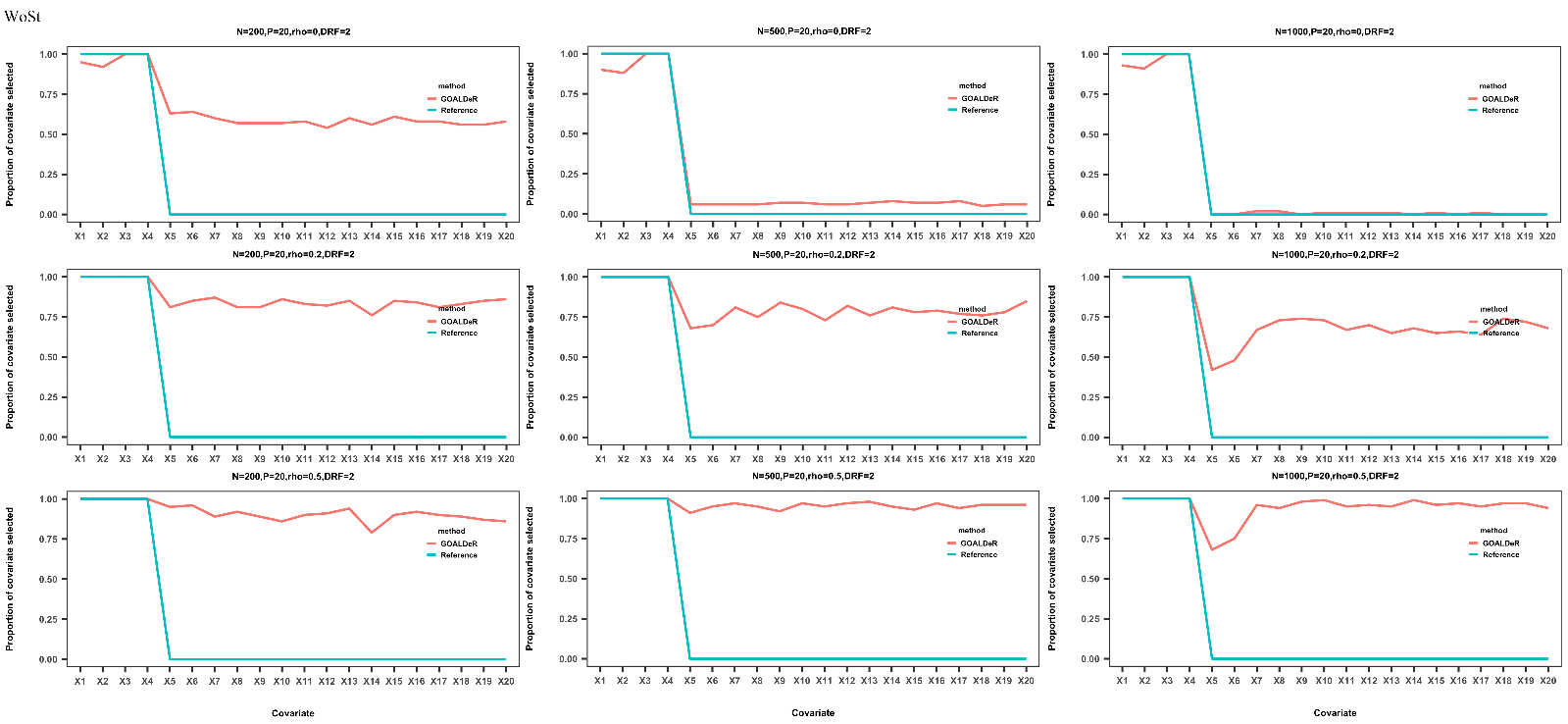


**Fig. S4.** Illustrations with a modest *p* = 20. The probability of covariate selection being balanced over 100 simulations under the setting where the confounders were relatively weakly correlated with the outcome (WoSt) and $\eta=2$.

In Figs. S5 to S7, we show the percentage of each covariate being selected for all three settings under Scenario 2. In the CoMt setting, GOALDeR selected confounders and prognostic covariates with a proportion of 1, but the proportion of selecting IVs and spurious covariates was also close to 1, regardless of sample size (Fig. S5). In the MoCt setting, GOALDeR excluded nearly all IVs and spurious covariates, but the selection of prognostic variables and some confounders was not satisfactory (Fig. S6). Fortunately, GOALDeR still provided nearly unbiased estimates across all sample sizes in the CoMt and MoCt settings (Fig. 2 in the main manuscript). In the MoMt setting, GOALDeR selected nearly all types of covariates (Fig. S7).


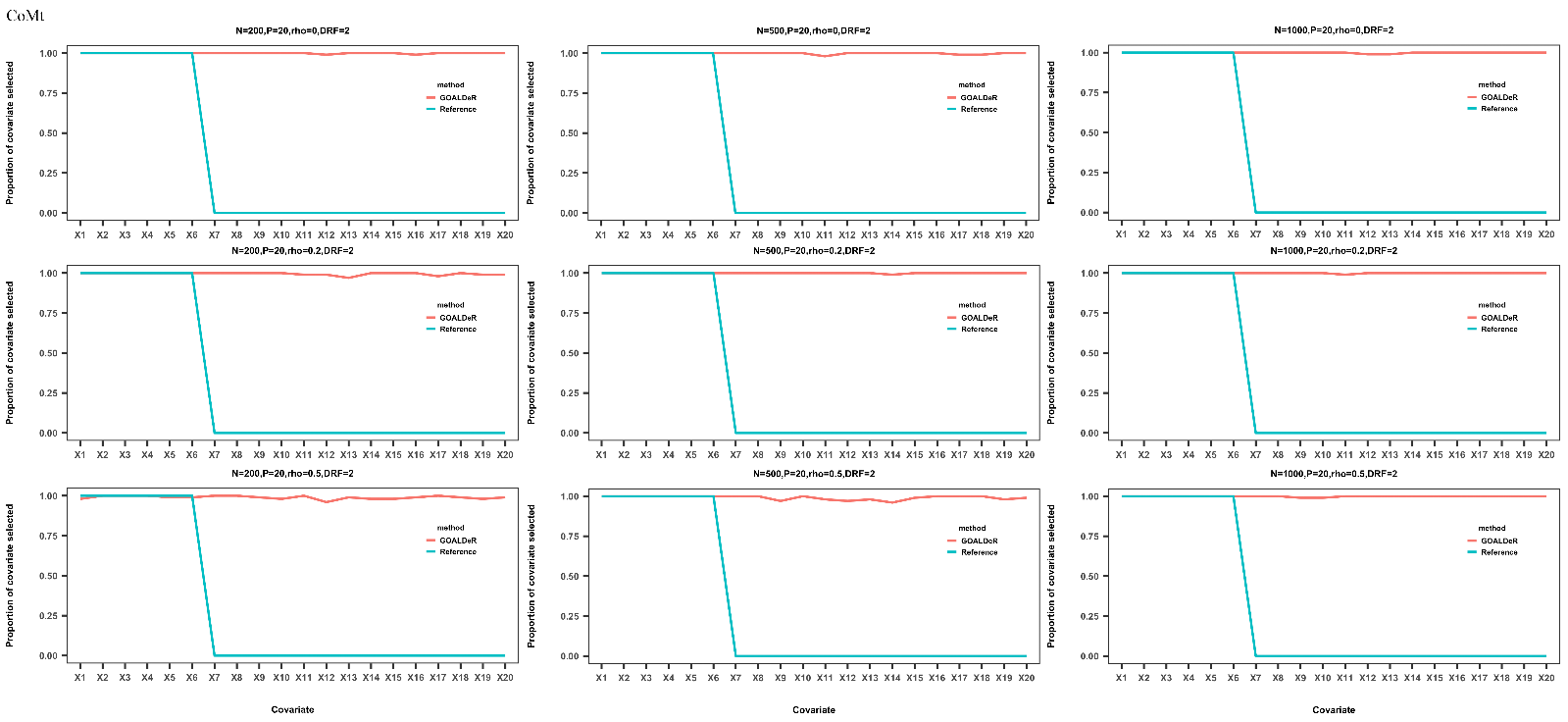


**Fig. S5.** Illustrations with a modest *p* = 20. The probability of covariate selection being balanced over 100 simulations under the setting where the outcome model was correctly specified and the GPS model was misspecified (CoMt) and $\eta=2$.


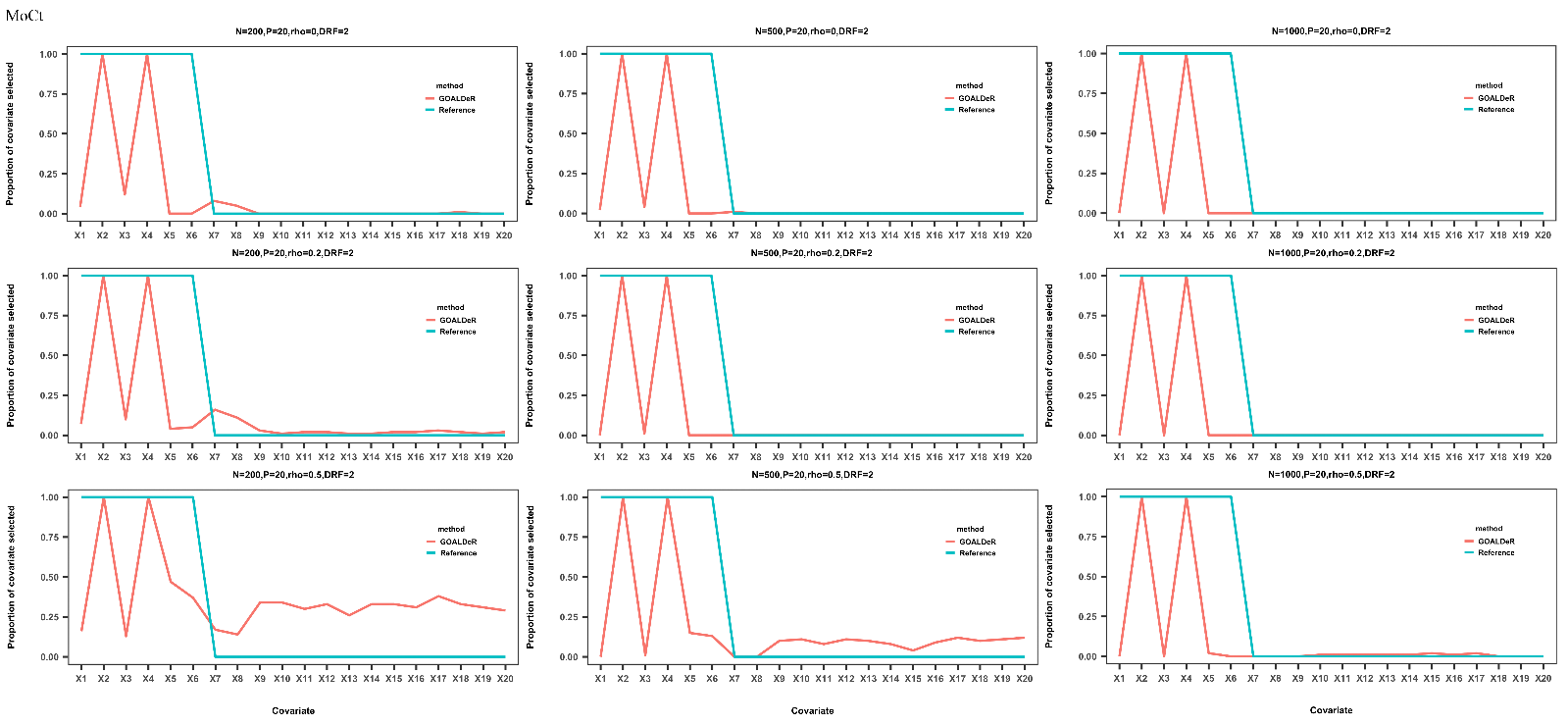


**Fig. S6.** Illustrations with a modest *p* = 20. The probability of covariate selection being balanced over 100 simulations under the setting where the outcome model was incorrectly specified and the GPS model was correctly specified (MoCt) and $\eta=2$.


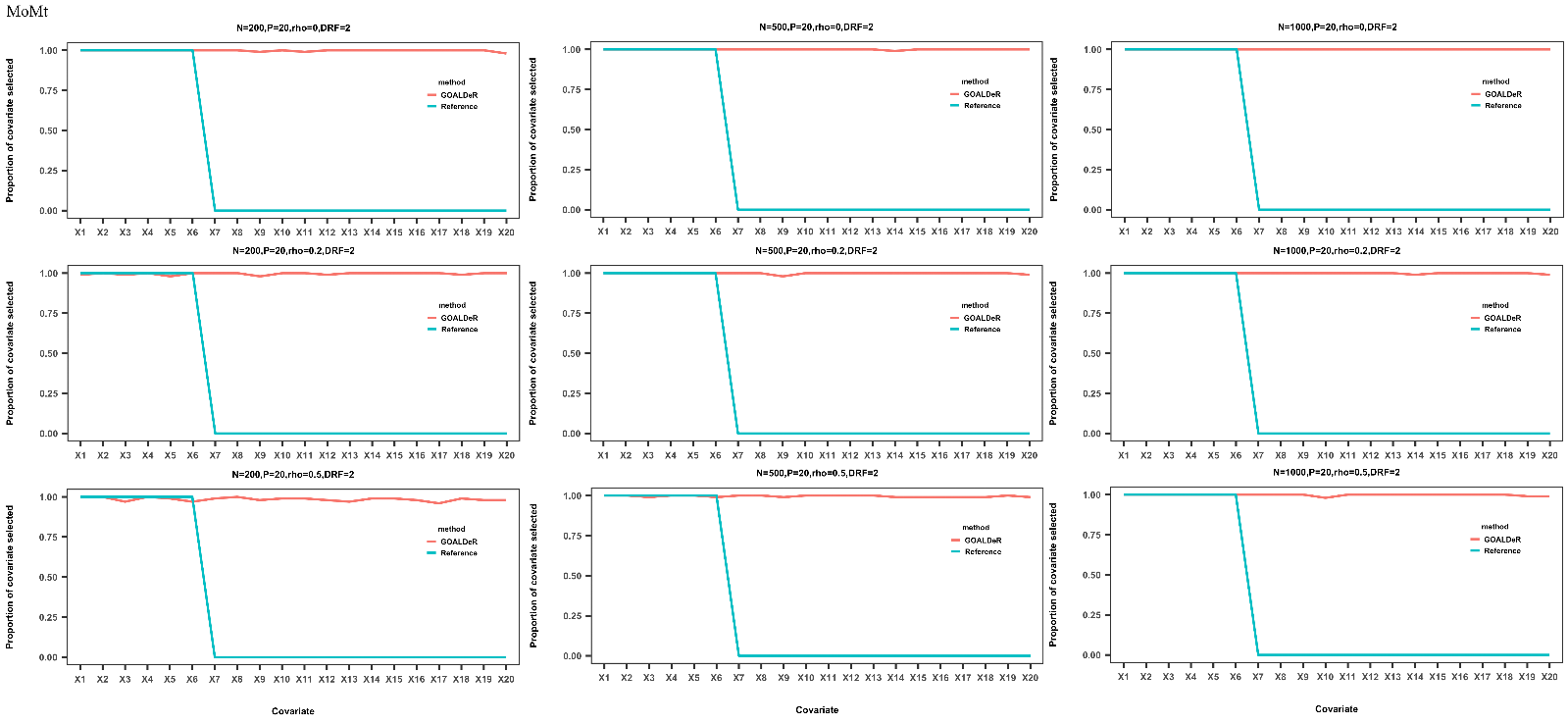


**Fig. S7.** Illustrations with a modest *p* = 20. The probability of covariate selection being balanced over 100 simulations under the setting where neither the GPS model nor the outcome model was correctly specified (MoMt) and $\eta=2$.

## 1.3 Estimation and variable selection with η = 0 and a modest *p* = 20

In Scenario 1 with η = 0, the boxplot of causal parameter estimates and the percentage of each covariate being selected are shown in Figs. S8 to S11. We found that GOALDeR performed similarly for η = 0 and η = 2, except in the setting of WoSt. In WoSt, GOALDeR may have underselected confounders (Fig. S11), but it still produced nearly unbiased estimates (Fig. S8).

In Figs. S12 to S15, we report the results of η = 0 under Scenario 2. These are similar to η = 2.


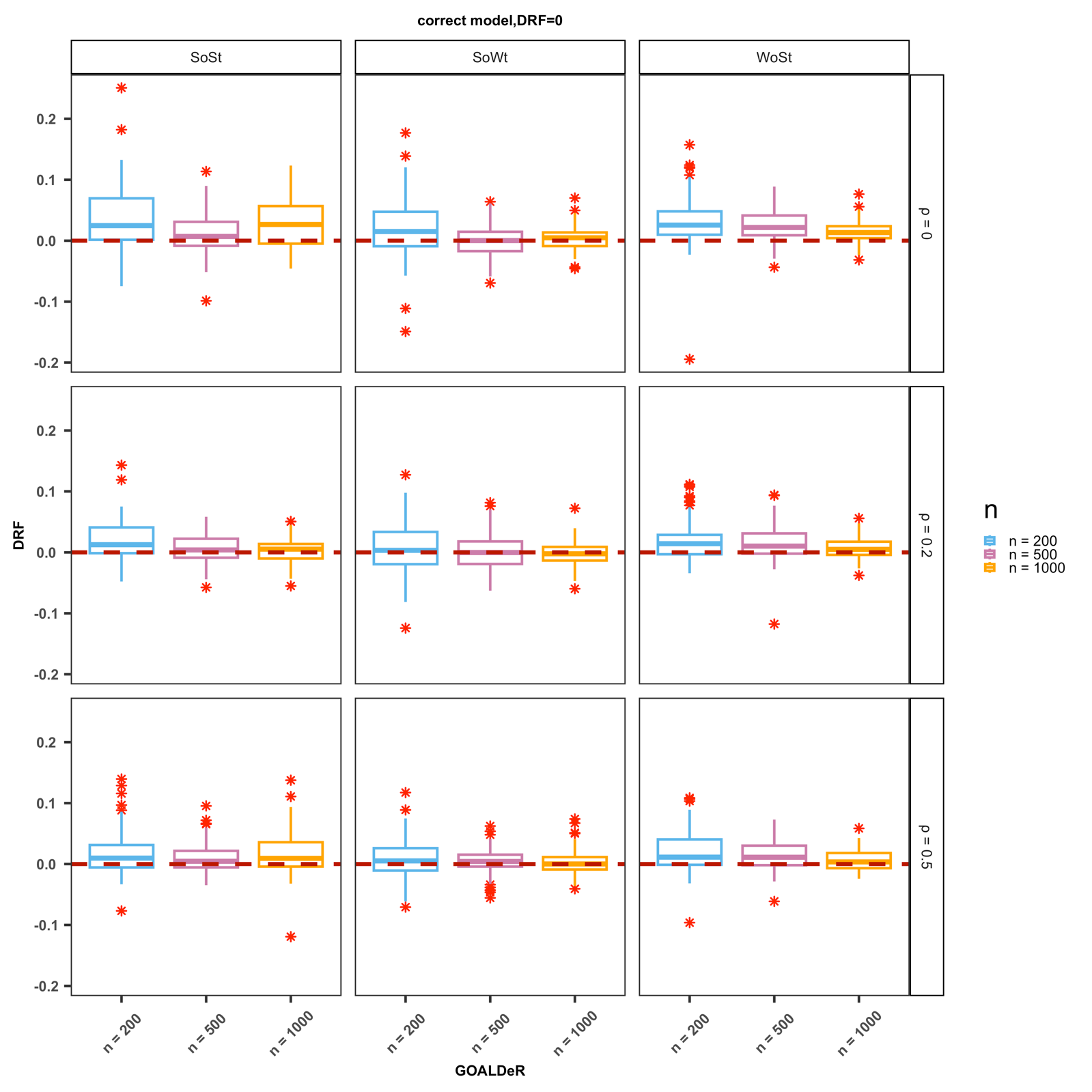


**Fig. S8.** Illustrations with a modest *p* = 20. Boxplot of parameters for the dose–response function (DRF) under Scenario 1 with η = 0. The true causal parameter of 0 is indicated by a dotted line, and the asterisks represent outliers.


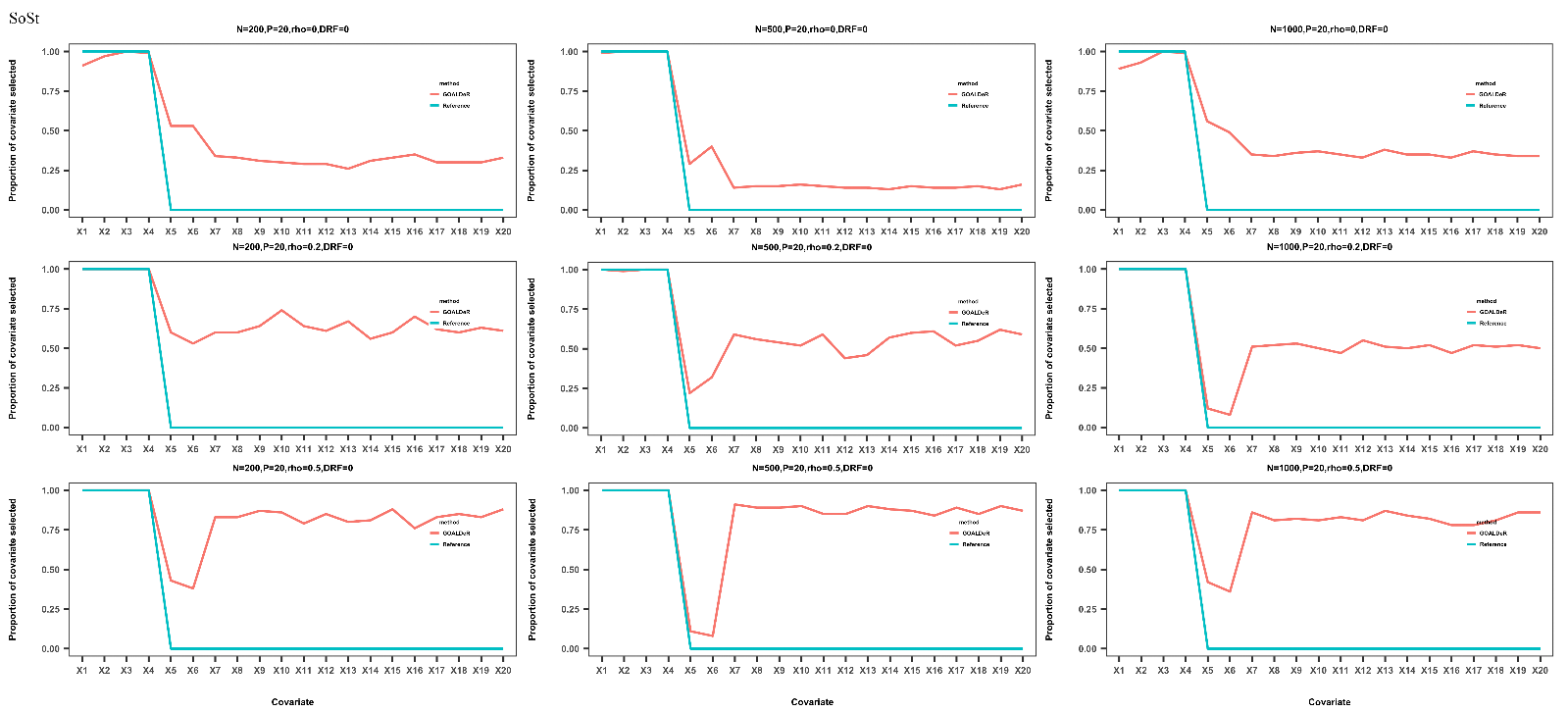


**Fig. S9.** Illustrations with a modest *p* = 20. The probability of covariate selection being balanced over 100 simulations under the setting where the confounders were strongly correlated with both the treatment and the outcome (SoSt) and $\eta=0$.


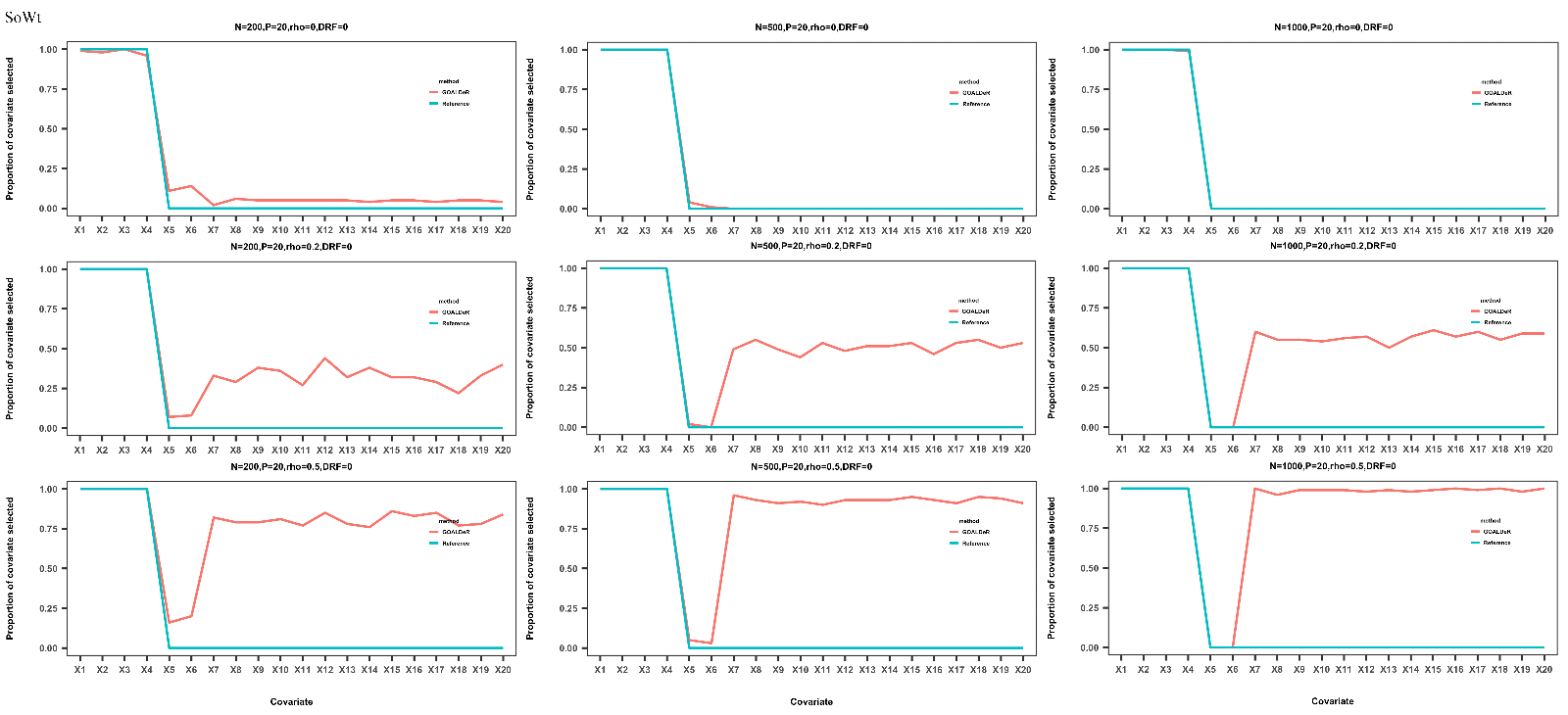


**Fig. S10.** Illustrations with a modest *p* = 20. The probability of covariate selection being balanced over 100 simulations under the setting where the confounders were relatively weakly correlated with the treatment (SoWt) and $\eta=0$.


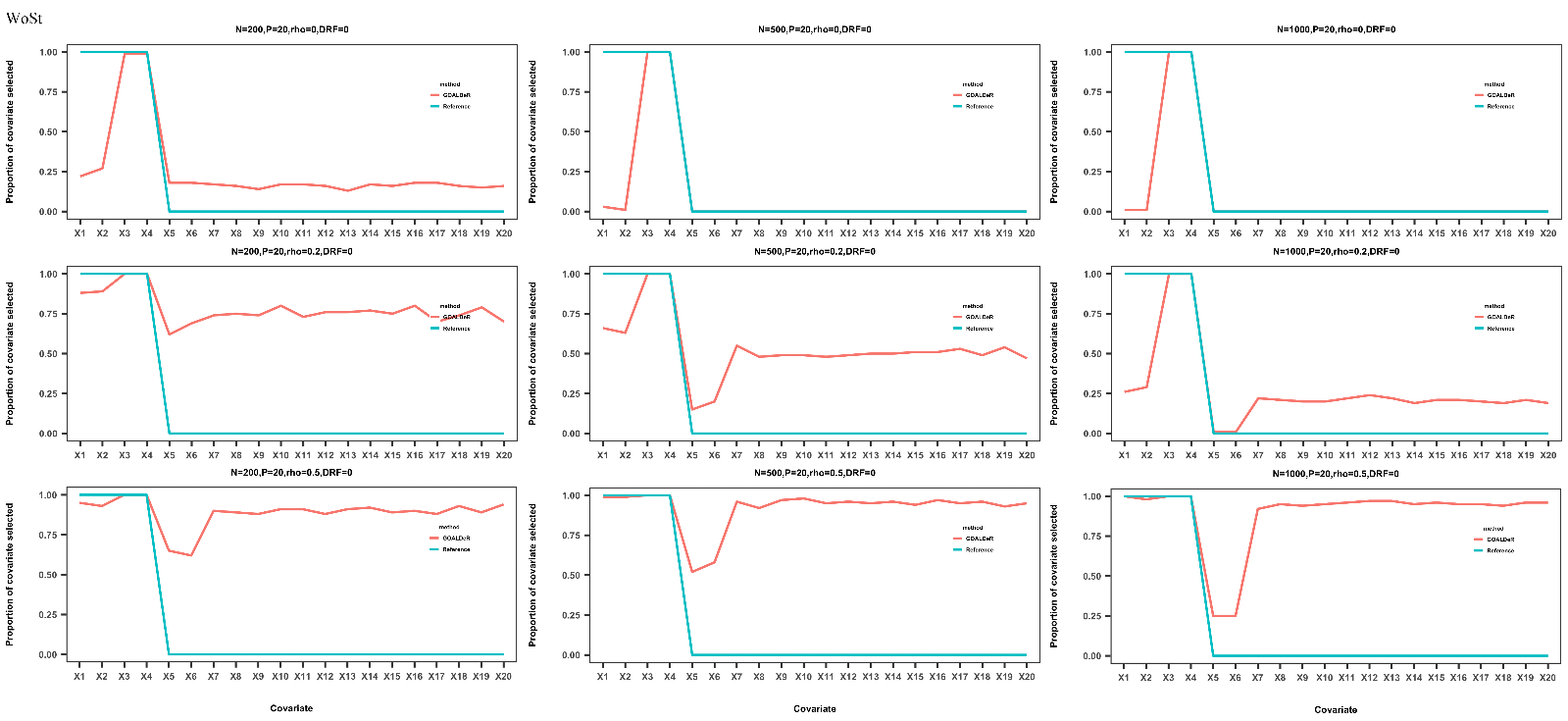


**Fig. S11.** Illustrations with a modest *p* = 20. The probability of covariate selection being balanced over 100 simulations under the setting where the confounders were relatively weakly correlated with the outcome (WoSt) and $\eta=0$.


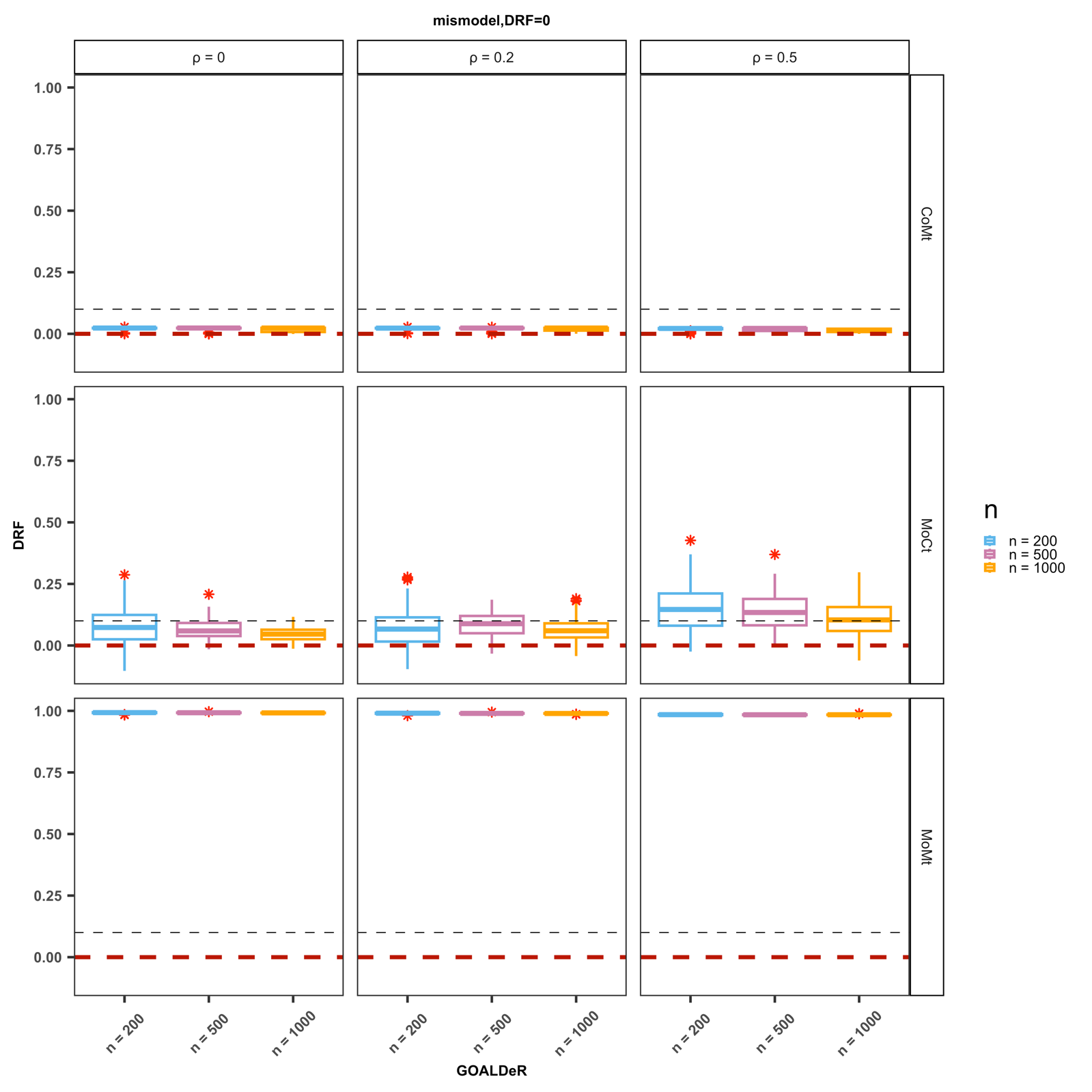


**Fig. S12** Illustrations with a modest *p* = 20. Boxplot of parameters for the dose–response function (DRF) under Scenario 2 with η = 0. The true causal parameter of 0 is indicated by a red dotted line, and the asterisks represent outliers. The black dashed line at 0.1 is primarily intended to assist in evaluation.


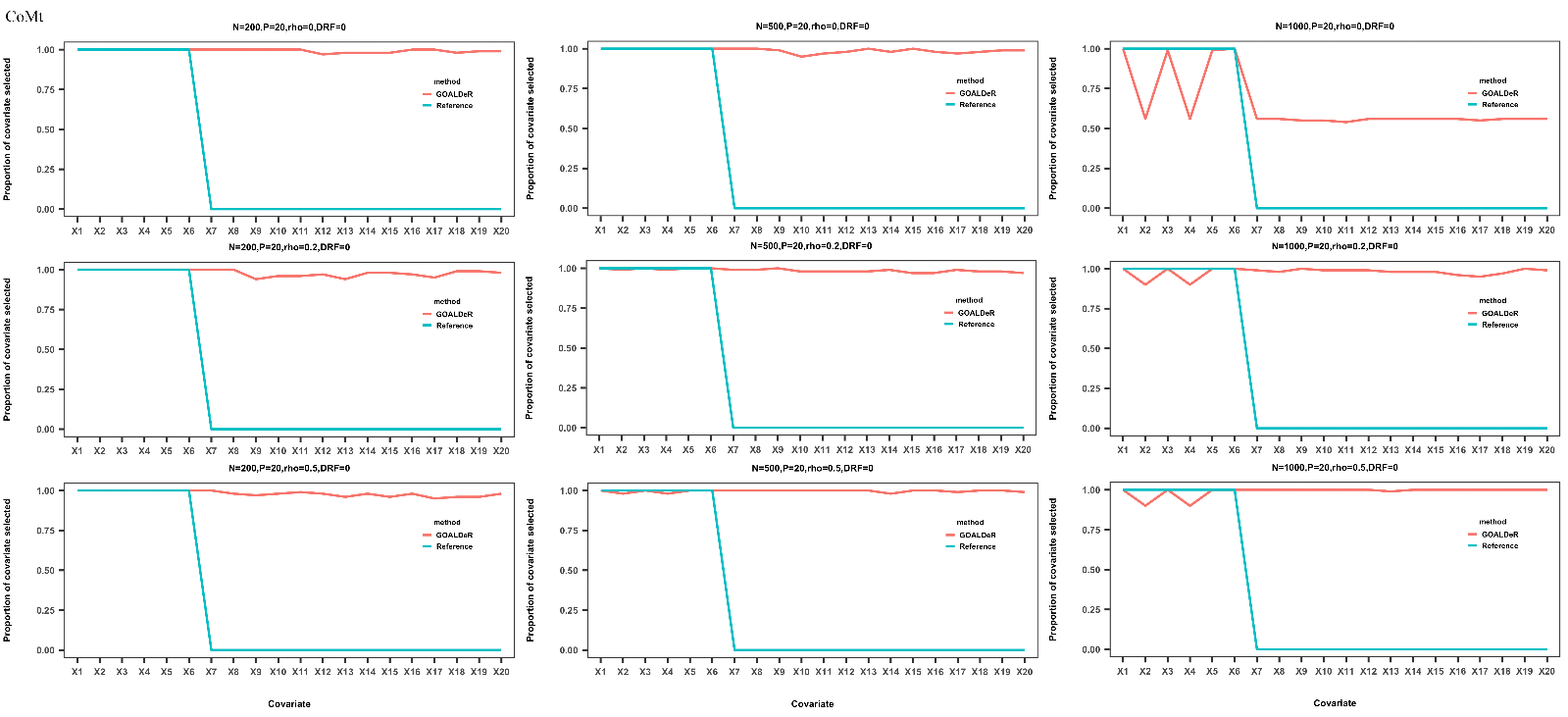


**Fig. S13.** Illustrations with a modest *p* = 20. The probability of covariate selection being balanced over 100 simulations under the setting where the outcome model was correctly specified and the GPS model was misspecified (CoMt) and $\eta=0$.


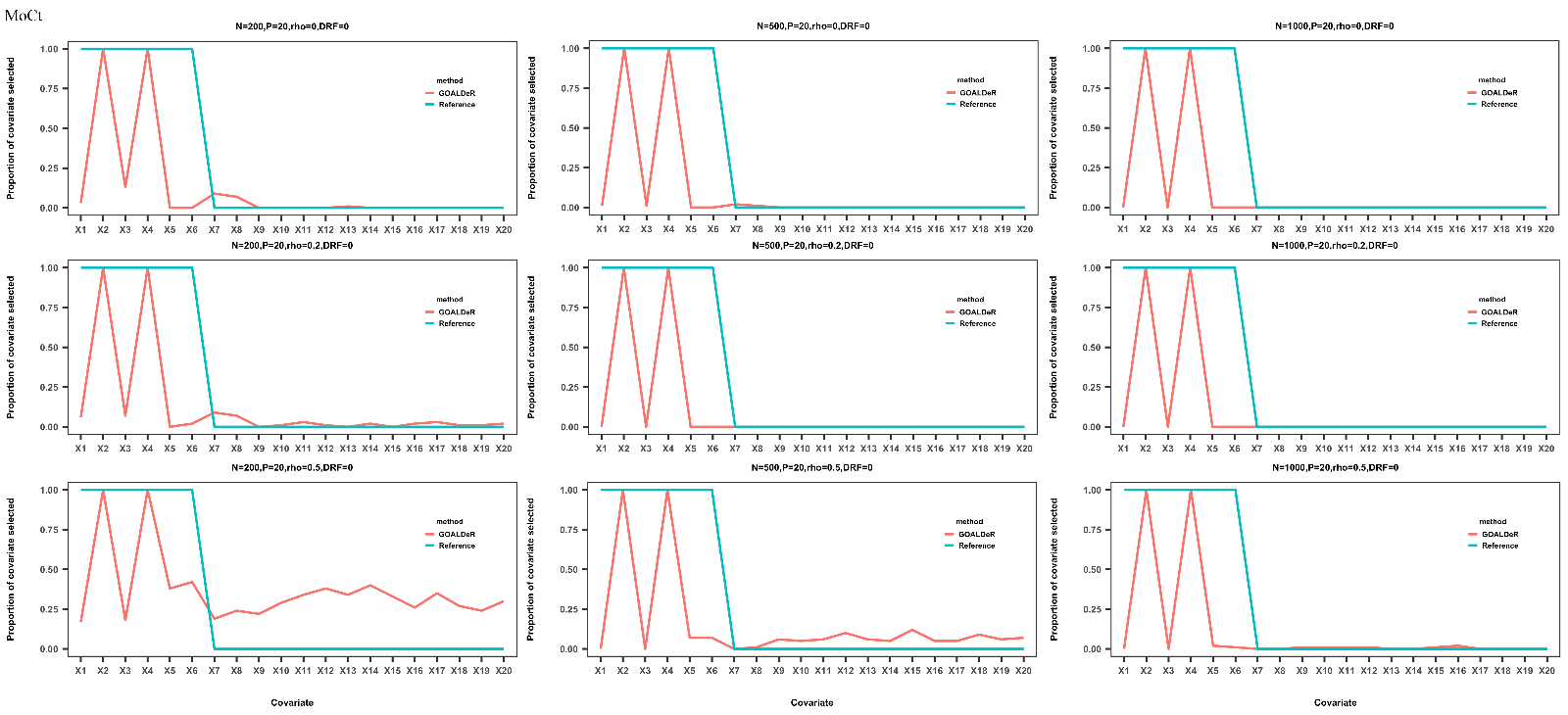


**Fig. S14.** Illustrations with a modest *p* = 20. The probability of covariate selection being balanced over 100 simulations under the setting where the outcome model was incorrectly specified and the GPS model was correctly specified (MoCt) and $\eta=$0.


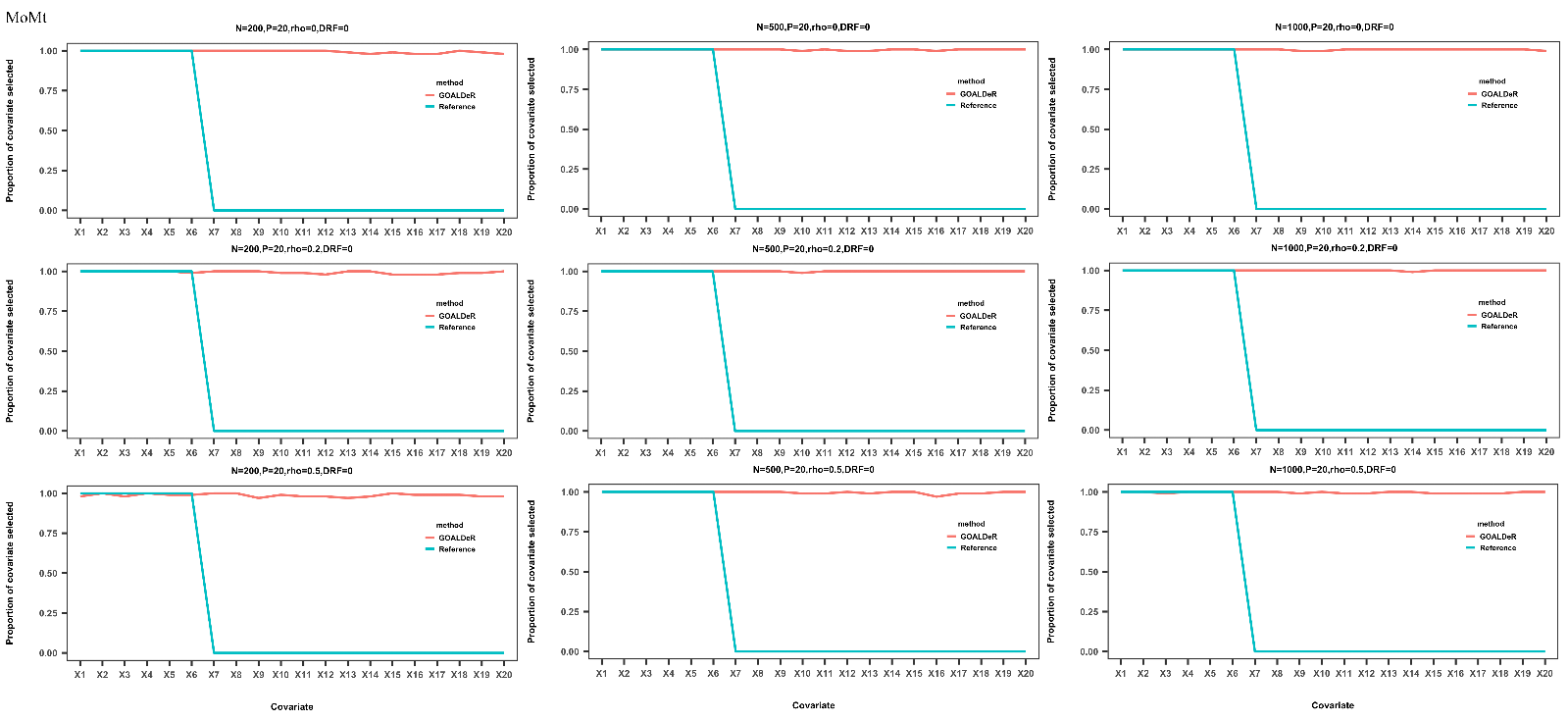


**Fig. S15.** Illustrations with a modest *p* = 20. The probability of covariate selection being balanced over 100 simulations under the setting where neither the GPS model nor the outcome model was correctly specified (MoMt), and $\eta=0$.

## 2. Results for η = 0 and a large number of covariates

## 2.1 Estimation and testing under Scenario 1 with η = 0 and with a large number of covariates

In Scenario 1, we compared the accuracy and precision of causal parameter estimates. Fig. S16 and Table S1 illustrate the results of Scenario 1. As with η = 2, GOALDeR provided nearly unbiased estimates and was robust to the *n*/*p* ratio and correlation between covariates. In contrast, the bias and variability (RMSE and empirical standard error) of GOAL became large as the correlation between covariates increased and the *n*/*p* ratio decreased. SL-DR provided similar estimation accuracy to GOALDeR, but the variability of its estimates was significantly larger than that of GOALDeR. The reason is presumably owing to ignoring the negative effects of IVs when fitting the GPS model.

**Table S1 is shown here, and we have attached it at the end of this file.**


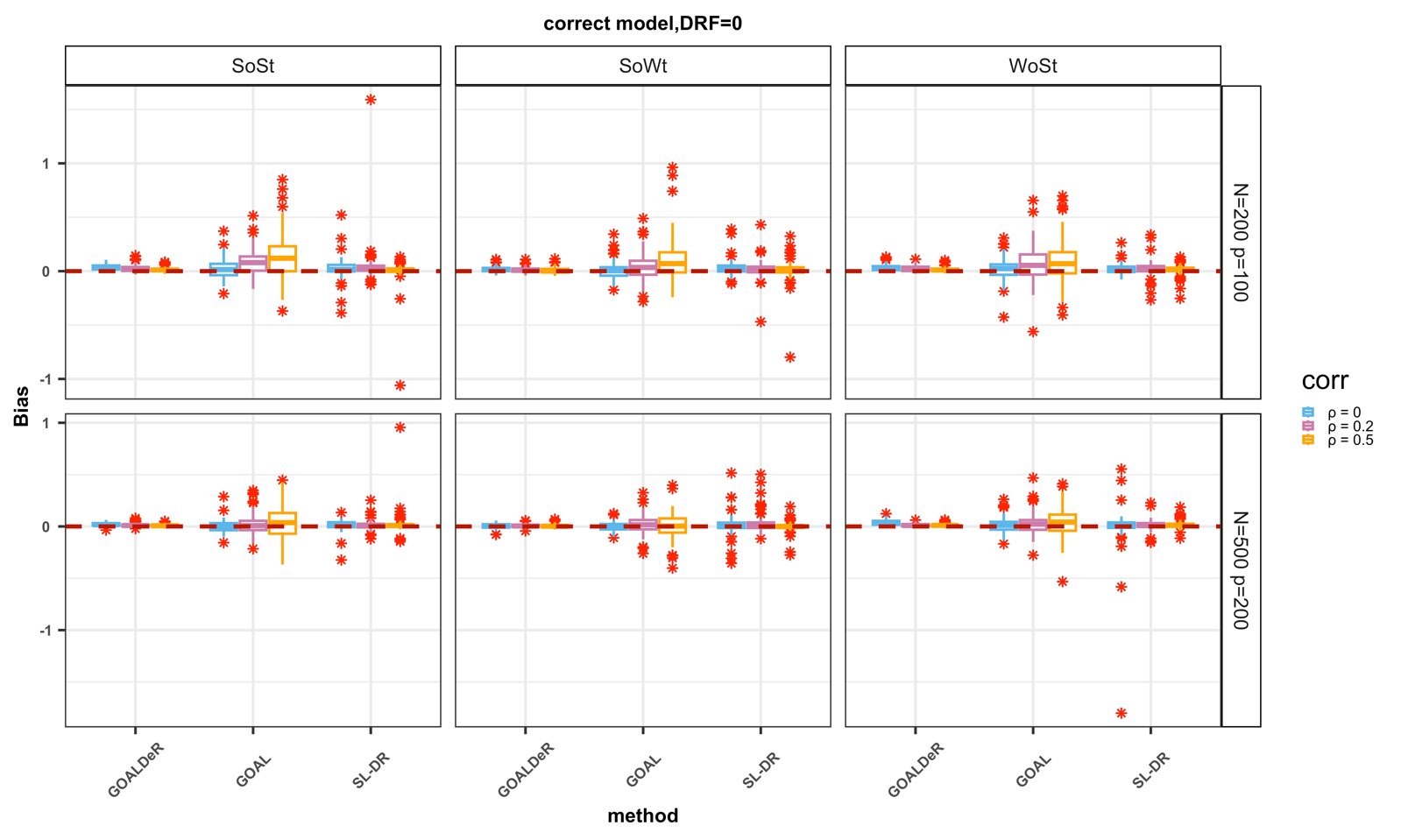


**Fig. S16.** Boxplot of the bias for causal parameters in the dose–response function (DRF) by our method and GOAL, SL-DR under Scenario 1 with η = 0. The zero reference line is indicated by a dotted line, and the asterisks represent outliers.

For GOALDeR, the estimated standard deviations (SDs) were close to the empirical standard errors in most cases, and half of the corresponding coverage of the 95% confidence interval (CI) was around 95%. One minus the coverage of the 95%CI is type I error when $\eta=0$, and GOALDeR was preferable for producing correctly controlled type I error when there was a strong correlation between covariates or when the confounders were relatively weakly correlated with the treatment. The bootstrap SDs were slightly higher than the empirical standard errors in most cases, and the corresponding type I error tended to be correctly controlled when there were correlations between covariates. For SL-DR, as with η = 2, the estimated SDs were significantly lower than the empirical standard errors, and the coverage of the 95% CI was consistently lower than that of the GOALDeR method. For GOAL, the estimated SDs were larger than the empirical standard errors, resulting in the coverage of the 95% CI tending to be conservative.

**2.2 Estimation and testing under Scenario 2 with η = 0 and with a large number of covariates**

In Scenario 2, we assessed the double robustness of GOALDeR, GOAL, and SL-DR. Fig. S17 and Table S2 illustrate the results of Scenario 2. As with η = 2, GOALDeR provided nearly unbiased estimates as long as one of the GPS and outcome models was correctly specified, and the biases were slightly impacted by the correlation between covariates and the n/p ratio. This indicates the double robustness of GOALDeR. In the setting of MoCt, the RMSE of the estimates by GOALDeR became large when there was a strong correlation between covariates. The SL-DR also tended double robustness, with biases similar to those of GOALDeR; however, the RMSE and the empirical standard errors of its estimates were larger than those of GOALDeR. In contrast, GOAL, which relies on a linear outcome model, became biased when the outcome model was misspecified (MoCt). In the MoMt setting, all three approaches were biased, with SL-DR exhibiting the largest biases and RMSE.

**Table S2 is shown here, and we have attached it at the end of this file.**

When one of the models was correctly specified (CoMt and MoCt), the estimated SDs of GOALDeR and SL-DR were less than or equal to the empirical standard errors, resulting in an overly inflated type I error in all three settings. The bootstrap SDs of GOALDeR were slightly higher than the empirical standard errors, and the type I error was nearly correctly controlled in the MoCt setting. For the GOAL method, the estimated SDs were slightly less than the empirical standard errors in the setting of CoMt, and the corresponding coverage was less than 95%. In the setting of MoCt, the estimated SDs of GOAL were significantly larger than the empirical standard errors, and the coverage was conservative (>95%). In the setting of MoMt, the coverages of all three methods were 0 because of the large bias.


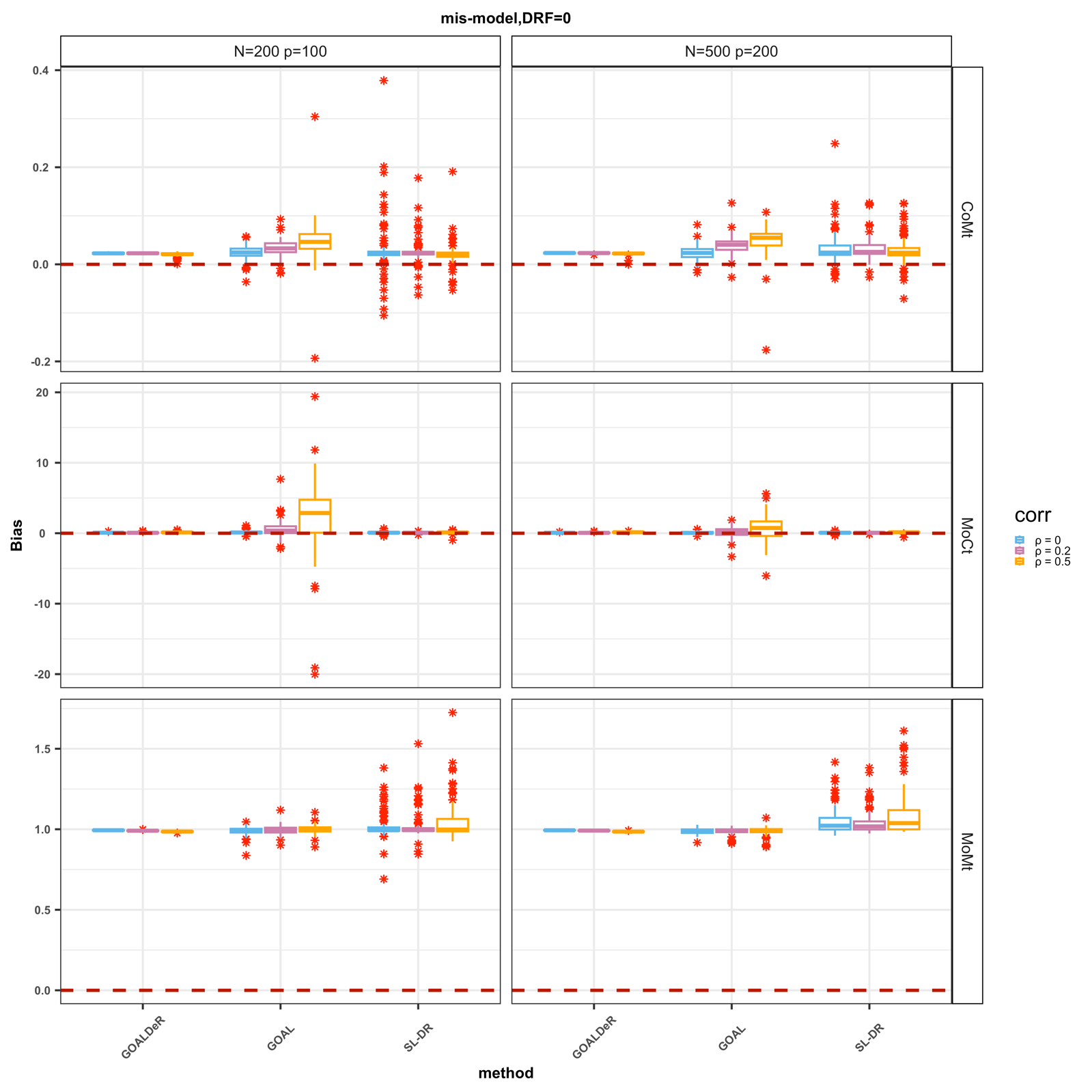


**Fig. S17.** Boxplot of the bias for the causal parameters in the dose–response function (DRF) using our method and GOAL, and SL-DR under Scenario 2 with η = 0. The zero reference line is indicated by a dotted line, and the asterisks represent outliers.

## 3. Results for η = 0.4, 0.7, and a large number of covariates

## 3.1 Estimation and testing under Scenario 1 with η = 0.4, 0.7, and with a large number of covariates

The summary statistics for Scenario 1 are listed in Table S3 and Table S4. The results of the estimates for *η* = 0.4 and 0.7 are similar to those for *η* = 2. GOALDeR showed nearly unbiased estimates across all three settings. The RMSE and the empirical standard error of GOALDeR were slightly decreased when *n* = 500 compared with *n* = 200. The accuracy and precision of GOALDeR were slightly impacted by the correlations between covariates. In contrast, as previously observed, the bias and variability (RMSE and empirical standard errors) of GOAL increased as the correlations between covariates increased and the *n*/*p* ratio decreased. Compared to GOALDeR, SL-DR provided similar estimation accuracy, but the precision was significantly worse than that of GOALDeR. The reason is presumably due to ignoring the negative effects of IVs when fitting the GPS model.

**Table S3 and S4 are shown here, and we have attached it at the end of this file.**

The results of the statistical testing were similar to those for $\eta=2$ except for the power. For GOALDeR, the estimated SDs were lower than the empirical standard errors in most cases, resulting in the coverage of the 95% confidence interval (CI) being less than 95%. For SL-DR, the estimated SDs were significantly lower than the empirical standard errors, and the coverage of the 95% CI was lower than that of the GOALDeR method. This implies that the estimated SDs for the GOALDeR and SL-DR methods were underestimated. For GOAL, the estimated SDs were larger than the empirical standard errors, resulting in the coverage of the 95% CI tending to be conservative. When the DRF parameter decreased from 2 to 0.4, the power for GOALDeR remained consistently at 1, while it slightly decreased for the SL-DR and significantly decreased for the GOAL method.

## 3.2 Estimation and testing under Scenario 2 with η = 0.4, 0.7, and with a large number of covariates

The summary statistics for Scenario 1 are listed in Table S5 and Table S6. The results of the estimates for *η* = 0.4 and 0.7 were similar to those for *η* = 2. GOALDeR yielded estimates that were close to the true value as long as one of the outcomes and GPS models were correctly specified, and the biases were less impacted by the correlation between covariates and the *n/p* ratio. This indicates the double robustness of GOALDeR. In the setting of MoCt, the variability (RMSE and empirical standard error) of the estimates by GOALDeR became large as the correlation between covariates increased, especially when the *n*/*p* ratio was small (*n*/*p* = 200/100). The SL-DR method also tended to be doubly robust. Compared to GOALDeR, SL-DR provided estimates with smaller biases and higher RMSE in the MoCt setting. However, when only the GPS model was non-linear (CoMt), its biases and RMSE were significantly larger than those of the GOALDeR method. In contrast, GOAL became biased when the outcome model was incorrectly specified because it relies on the assumption that the outcome model is linear for variable selection. In the MoMt setting, all three approaches were biased, with SL-DR exhibiting the largest biases and RMSE.

**Tables S5 and S6 are shown here, and we have attached it at the end of this file.**

The results of the statistical testing for *η* = 0.4 and 0.7 were similar to those for *η* = 2. When one of the models was correctly specified (CoMt and MoCt), the estimated SDs of GOALDeR were less than or equal to the empirical standard errors, resulting in the coverage being less than 95% in most cases. For the SL-DR method, the estimated SDs were significantly less than the empirical standard errors, and the coverage of the 95% CI was consistently lower than that of the GOALDeR method in the setting of MoCt. For the GOAL method, the estimated SDs were slightly less than empirical standard errors in the setting of CoMt, and the corresponding coverage was less than 95%. In the setting of MoCt, the estimated SDs were significantly larger than the empirical standard errors, and the coverage was conservative. In the setting of MoMt, the coverages of all three methods were 0 because of the large bias. When the DRF parameter decreased from 2 to 0.4, the power for GOALDeR remained consistently at 1, while it slightly decreased for SL-DR.

## The results for real data


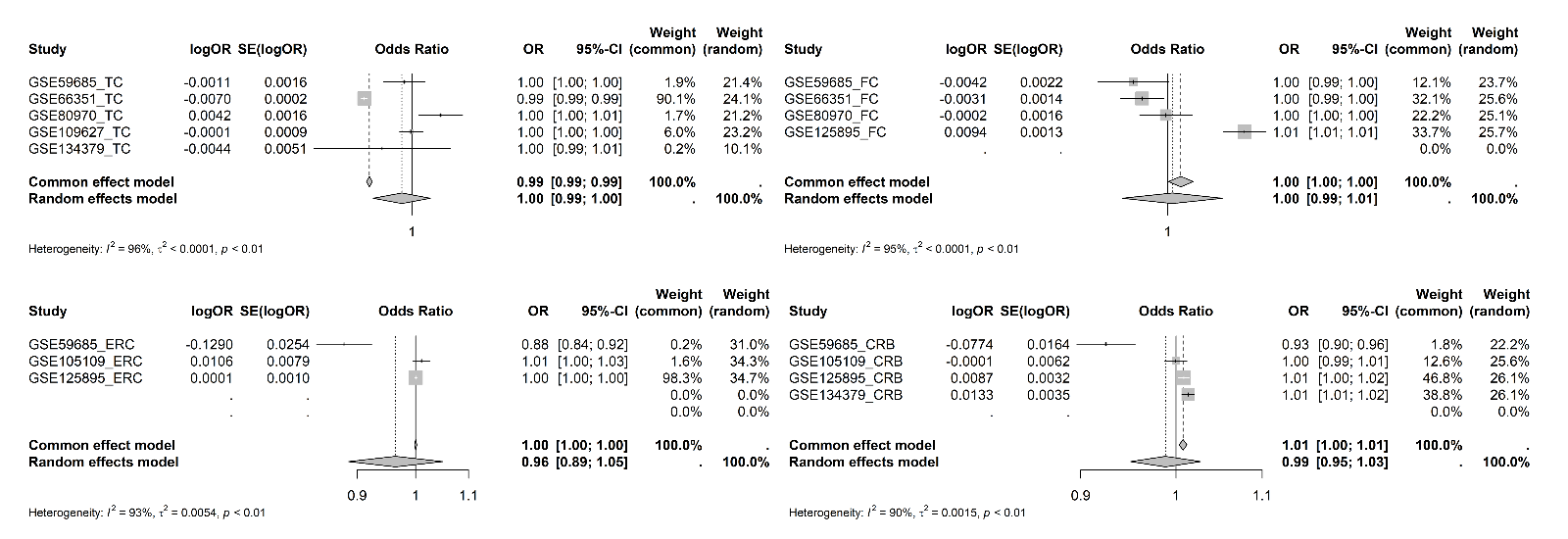


**Fig. S18** Forest plot of the SL-DR method for parameters in the dose–response function (DRF) of epigenetic age acceleration on Alzheimer’s disease in the frontal cortex (FC), temporal cortex (TC), entorhinal cortex (ERC), and cerebellum (CRB); OR, odds ratio; CI, confidence interval.

**Reference**

1. Gao Q, Zhang Y, Liang J, Sun H, Wang T: **High-dimensional generalized propensity score with application to omics data**. *Briefings in Bioinformatics* 2021, **22**(6):bbab331.

**Table S1** Summary statistics of the performance under Scenario 1 with the true parameter of DRF = 0

|  |  | **N=500, P=200** | | | | | | | | | **N=200, P=100** | | | | | | | | |
| --- | --- | --- | --- | --- | --- | --- | --- | --- | --- | --- | --- | --- | --- | --- | --- | --- | --- | --- | --- |
|  |  | **DRF** | **Est_** | **Est_** | **Est_** | **boot_** | **boot_** | **boot_** | **emp_** | **RMSE** | **DRF** | **Est_** | **Est_** | **Est_** | **boot_** | **boot_** | **boot_** | **emp_** | **RMSE** |
|  |  |  | **Std** | **Coverage** | **Type I error** | **std** | **coverage** | **Type I error** | **std** |  |  | **Std** | **Coverage** | **Type I error** | **std** | **coverage** | **type I error** | **std** |  |
| SoSt  rho=0 | GOALDeR | 0.019 | 0.023 | 0.85 | 0.15 | 0.026 | 0.96 | 0.04 | 0.019 | 0.027 | 0.036 | 0.032 | 0.81 | 0.19 | 0.034 | 0.9 | 0.1 | 0.03 | 0.046 |
|  | SL-DR | 0.01 | 0.03 | 0.82 | 0.18 |  |  |  | 0.05 | 0.051 | 0.024 | 0.043 | 0.78 | 0.22 |  |  |  | 0.091 | 0.094 |
|  | GOAL | -0.001 | 0.1 | 0.99 | 0.01 |  |  |  | 0.056 | 0.056 | 0.021 | 0.16 | 0.99 | 0.01 |  |  |  | 0.094 | 0.096 |
| SoWt  rho=0 | GOALDeR | 0.003 | 0.026 | 0.97 | 0.03 | 0.014 | 0.74 | 0.26 | 0.025 | 0.025 | 0.018 | 0.039 | 0.96 | 0.04 | 0.024 | 0.95 | 0.05 | 0.025 | 0.031 |
|  | SL-DR | 0.011 | 0.046 | 0.89 | 0.11 |  |  |  | 0.096 | 0.096 | 0.029 | 0.052 | 0.88 | 0.12 |  |  |  | 0.069 | 0.074 |
|  | GOAL | -0.002 | 0.089 | 1 | 0 |  |  |  | 0.044 | 0.044 | 0.007 | 0.15 | 1 | 0 |  |  |  | 0.079 | 0.079 |
| WoSt  rho=0 | GOALDeR | 0.036 | 0.022 | 0.62 | 0.38 | 0.021 | 0.64 | 0.36 | 0.026 | 0.045 | 0.031 | 0.033 | 0.81 | 0.19 | 0.026 | 0.81 | 0.19 | 0.031 | 0.044 |
|  | SL-DR | -0.003 | 0.048 | 0.81 | 0.19 |  |  |  | 0.209 | 0.208 | 0.021 | 0.029 | 0.78 | 0.22 |  |  |  | 0.048 | 0.052 |
|  | GOAL | 0.008 | 0.087 | 0.95 | 0.05 |  |  |  | 0.072 | 0.072 | 0.02 | 0.14 | 0.96 | 0.04 |  |  |  | 0.101 | 0.102 |
| SoSt  rho=0.2 | GOALDeR | 0.013 | 0.021 | 0.92 | 0.08 | 0.03 | 0.99 | 0.01 | 0.017 | 0.021 | 0.027 | 0.031 | 0.84 | 0.16 | 0.038 | 0.95 | 0.05 | 0.032 | 0.042 |
|  | SL-DR | 0.013 | 0.027 | 0.85 | 0.15 |  |  |  | 0.045 | 0.046 | 0.041 | 0.044 | 0.76 | 0.24 |  |  |  | 0.165 | 0.169 |
|  | GOAL | 0.021 | 0.137 | 0.98 | 0.02 |  |  |  | 0.103 | 0.105 | 0.076 | 0.183 | 0.99 | 0.01 |  |  |  | 0.118 | 0.14 |
| SoWt  rho=0.2 | GOALDeR | 0.006 | 0.024 | 0.98 | 0.02 | 0.018 | 0.94 | 0.06 | 0.018 | 0.019 | 0.01 | 0.038 | 0.99 | 0.01 | 0.028 | 0.99 | 0.01 | 0.024 | 0.026 |
|  | SL-DR | 0.03 | 0.046 | 0.89 | 0.11 |  |  |  | 0.093 | 0.097 | 0.016 | 0.052 | 0.88 | 0.12 |  |  |  | 0.078 | 0.08 |
|  | GOAL | 0.003 | 0.174 | 0.99 |  |  |  |  | 0.12 | 0.119 | 0.043 | 0.206 | 0.99 | 0.01 |  |  |  | 0.128 | 0.134 |
| WoSt  rho=0.2 | GOALDeR | 0.012 | 0.021 | 0.91 | 0.09 | 0.021 | 0.95 | 0.05 | 0.017 | 0.021 | 0.025 | 0.03 | 0.87 | 0.13 | 0.026 | 0.89 | 0.11 | 0.029 | 0.038 |
|  | SL-DR | 0.011 | 0.032 | 0.86 | 0.14 |  |  |  | 0.048 | 0.049 | 0.024 | 0.04 | 0.76 | 0.24 |  |  |  | 0.071 | 0.075 |
|  | GOAL | 0.023 | 0.125 | 0.99 | 0.01 |  |  |  | 0.102 | 0.104 | 0.069 | 0.16 | 0.91 | 0.09 |  |  |  | 0.159 | 0.172 |
| SoSt  rho=0.5 | GOALDeR | 0.009 | 0.024 | 0.98 | 0.02 | 0.027 | 1 | 0 | 0.013 | 0.016 | 0.013 | 0.031 | 0.96 | 0.04 | 0.032 | 0.99 | 0.01 | 0.02 | 0.024 |
|  | SL-DR | 0.021 | 0.033 | 0.93 | 0.07 |  |  |  | 0.103 | 0.104 | 0 | 0.03 | 0.84 | 0.16 |  |  |  | 0.115 | 0.114 |
|  | GOAL | 0.035 | 0.183 | 0.95 | 0.05 |  |  |  | 0.158 | 0.161 | 0.136 | 0.221 | 0.88 | 0.12 |  |  |  | 0.209 | 0.248 |
| SoWt  rho=0.5 | GOALDeR | 0.004 | 0.028 | 0.99 | 0.01 | 0.018 | 0.94 | 0.06 | 0.016 | 0.016 | 0.009 | 0.039 | 0.96 | 0.04 | 0.027 | 0.96 | 0.04 | 0.029 | 0.03 |
|  | SL-DR | -0.001 | 0.033 | 0.96 | 0.04 |  |  |  | 0.051 | 0.051 | 0.011 | 0.052 | 0.88 | 0.12 |  |  |  | 0.106 | 0.106 |
|  | GOAL | 0.003 | 0.174 | 0.99 | 0.01 |  |  |  | 0.12 | 0.119 | 0.1 | 0.25 | 0.97 | 0.03 |  |  |  | 0.187 | 0.211 |
| WoSt  rho=0.5 | GOALDeR | 0.012 | 0.023 | 0.96 | 0.04 | 0.019 | 0.96 | 0.04 | 0.014 | 0.018 | 0.016 | 0.031 | 0.93 | 0.07 | 0.024 | 0.96 | 0.04 | 0.023 | 0.028 |
|  | SL-DR | 0.015 | 0.033 | 0.87 | 0.13 |  |  |  | 0.036 | 0.039 | 0.012 | 0.026 | 0.82 | 0.18 |  |  |  | 0.048 | 0.049 |
|  | GOAL | 0.037 | 0.161 | 0.94 | 0.06 |  |  |  | 0.138 | 0.142 | 0.098 | 0.199 | 0.89 | 0.11 |  |  |  | 0.206 | 0.228 |

Notations: DRF =0, the parameter of DRF is 0; rho=0, rho=0.2, and rho=0.5, there are no, moderate and strong correlations between covariates; emp_std: the empirical standard deviation of the estimates; Est_Std: the mean of standard deviation estimates using sandwich-type estimator (GOAL) or regression of the treatment on pseudo-outcome (GOALDeR and SL-DR); Boot_Std: the mean of bootstrapped standard deviation; coverage: coverage probability of the 95% confidence interval; Type I error: one minus coverage probability when $\eta=0$; RMSE: root mean square error calculated as $RMSE=\sqrt{\frac{1}{B}\sum_{b=1}^{B} \left( \hat{\eta}_{b}-\eta\right)^{2}}$.

**Table S2** Summary statistics of the performance under Scenario 2 with the true parameter of DRF = 0

|  |  | **N=500, P=200** | | | | | | | | | **N=200, P=100** | | | | | | | | |
| --- | --- | --- | --- | --- | --- | --- | --- | --- | --- | --- | --- | --- | --- | --- | --- | --- | --- | --- | --- |
|  |  | **DRF** | **Est_** | **Est_** | **Est_** | **boot_** | **boot_** | **boot_** | **emp_** | **RMSE** | **DRF** | **Est_**  **Std** | **Est_** | **Est_** | **boot_** | **boot_** | **boot_** | **emp_** | **RMSE** |
|  |  |  | **Std** | **Coverage** | **Type I error** | **std** | **coverage** | **Type I error** | **std** |  |  |  | **Coverage** | **Type I error** | **std** | **coverage** | **Type I error** | **std** |  |
| CoMt  rho=0 | GOALDeR | 0.023 | 0.001 | 0 | 1 | 0.002 | 0 | 1 | 0.001 | 0.023 | 0.023 | 0.001 | 0 | 1 | 0.002 | 0 | 1 | 0.002 | 0.023 |
|  | SL-DR | 0.032 | 0.006 | 0.09 | 0.91 |  |  |  | 0.033 | 0.046 | 0.03 | 0.007 | 0.11 | 0.89 |  |  |  | 0.054 | 0.062 |
|  | GOAL | 0.024 | 0.011 | 0.44 | 0.56 |  |  |  | 0.015 | 0.028 | 0.024 | 0.014 | 0.45 | 0.55 |  |  |  | 0.014 | 0.028 |
| MoCt  rho=0 | GOALDeR | 0.068 | 0.055 | 0.81 | 0.19 | 0.051 | 0.74 | 0.26 | 0.045 | 0.081 | 0.081 | 0.077 | 0.85 | 0.15 | 0.08 | 0.9 | 0.1 | 0.064 | 0.103 |
|  | SL-DR | 0.066 | 0.065 | 0.63 | 0.37 |  |  |  | 0.098 | 0.118 | 0.083 | 0.082 | 0.72 | 0.28 |  |  |  | 0.135 | 0.158 |
|  | GOAL | 0.063 | 2.275 | 1 | 0 |  |  |  | 0.169 | 0.179 | 0.116 | 3.146 | 1 | 0 |  |  |  | 0.23 | 0.256 |
| MoMt  rho=0 | GOALDeR | 0.994 | 0.002 | 0 | 1 | 0.003 | 0 | 1 | 0.001 | 0.994 | 0.994 | 0.003 | 0 | 1 | 0.004 | 0 | 1 | 0.003 | 0.994 |
|  | SL-DR | 1.051 | 0.016 | 0 | 1 |  |  |  | 0.08 | 1.054 | 1.017 | 0.013 | 0 | 1 |  |  |  | 0.08 | 1.02 |
|  | GOAL | 0.988 | 0.017 | 0 | 1 |  |  |  | 0.018 | 0.988 | 0.989 | 0.022 | 0 | 1 |  |  |  | 0.025 | 0.989 |
| CoMt  rho=0.2 | GOALDeR | 0.023 | 0.001 | 0 | 1 | 0.002 | 0 | 1 | 0.001 | 0.023 | 0.023 | 0.001 | 0 | 1 | 0.002 | 0 | 1 | 0.002 | 0.023 |
|  | SL-DR | 0.031 | 0.006 | 0.11 | 0.89 |  |  |  | 0.023 | 0.039 | 0.026 | 0.004 | 0.07 | 0.93 |  |  |  | 0.026 | 0.036 |
|  | GOAL | 0.039 | 0.015 | 0.21 | 0.79 |  |  |  | 0.017 | 0.043 | 0.034 | 0.017 | 0.32 | 0.68 |  |  |  | 0.016 | 0.037 |
| MoCt  rho=0.2 | GOALDeR | 0.062 | 0.046 | 0.75 | 0.25 | 0.06 | 0.87 | 0.13 | 0.052 | 0.08 | 0.074 | 0.061 | 0.77 | 0.23 | 0.089 | 0.97 | 0.03 | 0.075 | 0.105 |
|  | SL-DR | 0.06 | 0.057 | 0.58 | 0.42 |  |  |  | 0.075 | 0.095 | 0.075 | 0.054 | 0.6 | 0.4 |  |  |  | 0.084 | 0.112 |
|  | GOAL | 0.175 | 2.658 | 1 | 0 |  |  |  | 0.717 | 0.734 | 0.566 | 3.447 | 0.99 | 0.01 |  |  |  | 1.161 | 1.287 |
| MoMt  rho=0.2 | GOALDeR | 0.992 | 0.002 | 0 | 1 | 0.003 | 0 | 1 | 0.001 | 0.992 | 0.991 | 0.003 | 0 | 1 | 0.004 | 0 | 1 | 0.003 | 0.991 |
|  | SL-DR | 1.042 | 0.013 | 0 | 1 |  |  |  | 0.073 | 1.044 | 1.017 | 0.012 | 0 | 1 |  |  |  | 0.081 | 1.02 |
|  | GOAL | 0.989 | 0.016 | 0 | 1 |  |  |  | 0.02 | 0.989 | 0.994 | 0.02 | 0 | 1 |  |  |  | 0.026 | 0.994 |
| CoMt  rho=0.5 | GOALDeR | 0.022 | 0.001 | 0.01 | 0.99 | 0.003 | 0.02 | 0.98 | 0.003 | 0.022 | 0.02 | 0.001 | 0.02 | 0.98 | 0.004 | 0.04 | 0.96 | 0.004 | 0.021 |
|  | SL-DR | 0.029 | 0.006 | 0.1 | 0.9 |  |  |  | 0.028 | 0.041 | 0.02 | 0.004 | 0.11 | 0.89 |  |  |  | 0.024 | 0.031 |
|  | GOAL | 0.049 | 0.019 | 0.23 | 0.77 |  |  |  | 0.031 | 0.058 | 0.047 | 0.023 | 0.24 | 0.76 |  |  |  | 0.041 | 0.062 |
| MoCt  rho=0.5 | GOALDeR | 0.14 | 0.04 | 0.14 | 0.86 | 0.104 | 0.89 | 0.11 | 0.054 | 0.15 | 0.136 | 0.056 | 0.49 | 0.51 | 0.138 | 0.95 | 0.05 | 0.098 | 0.168 |
|  | SL-DR | 0.125 | 0.042 | 0.18 | 0.82 |  |  |  | 0.098 | 0.159 | 0.117 | 0.063 | 0.28 | 0.72 |  |  |  | 0.168 | 0.204 |
|  | GOAL | 0.706 | 3.358 | 1 | 0 |  |  |  | 1.802 | 1.927 | 2.184 | 4.623 | 0.85 | 0.15 |  |  |  | 4.901 | 5.343 |
| MoMt  rho=0.5 | GOALDeR | 0.986 | 0.002 | 0 | 1 | 0.004 | 0 | 1 | 0.002 | 0.986 | 0.985 | 0.003 | 0 | 1 | 0.006 | 0 | 1 | 0.003 | 0.985 |
|  | SL-DR | 1.09 | 0.021 | 0 | 1 |  |  |  | 0.135 | 1.099 | 1.05 | 0.018 | 0 | 1 |  |  |  | 0.118 | 1.056 |
|  | GOAL | 0.989 | 0.017 | 0 | 1 |  |  |  | 0.024 | 0.989 | 1 | 0.021 | 0 | 1 |  |  |  | 0.026 | 1 |

Notations: DRF =0, the parameter of DRF is 0; rho=0, rho=0.2, and rho=0.5, there are no, moderate and strong correlations between covariates; emp_std: the empirical standard deviation of the estimates; Est_Std: the mean of standard deviation estimates using sandwich-type estimator (GOAL) or regression of the treatment on pseudo-outcome (GOALDeR and SL-DR); Boot_Std: the mean of bootstrapped standard deviation; coverage: coverage probability of the 95% confidence interval; Type I error: one minus coverage probability when $\eta=0$; RMSE: root mean square error calculated as $RMSE=\sqrt{\frac{1}{B}\sum_{b=1}^{B} \left( \hat{\eta}_{b}-\eta\right)^{2}}$.

**Table S3** Summary statistics of the performance under Scenario 1 with the true parameter of DRF = 0.7

|  |  | **N=500, P=200** | | | | | | **N=200, P=100** | | | | | |
| --- | --- | --- | --- | --- | --- | --- | --- | --- | --- | --- | --- | --- | --- |
|  |  | **DRF** | **Est_**  **Std** | **Est_**  **Coverage** | **Est_**  **Power** | **emp_**  **std** | **RMSE** | **DRF** | **Est_**  **Std** | **Est_**  **Coverage** | **Est_**  **Power** | **emp_**  **std** | **RMSE** |
| SoSt  rho=0 | GOALDeR | 0.706 | 0.025 | 0.9 | 1 | 0.028 | 0.029 | 0.715 | 0.033 | 0.82 | 1 | 0.044 | 0.046 |
|  | SL-DR | 0.699 | 0.031 | 0.79 | 0.99 | 0.055 | 0.055 | 0.711 | 0.042 | 0.73 | 1 | 0.091 | 0.092 |
|  | GOAL | 0.699 | 0.1 | 0.99 | 1 | 0.056 | 0.056 | 0.721 | 0.16 | 0.99 | 0.99 | 0.094 | 0.096 |
| SoWt  rho=0 | GOALDeR | 0.695 | 0.027 | 0.94 | 1 | 0.029 | 0.029 | 0.689 | 0.04 | 0.89 | 1 | 0.044 | 0.045 |
|  | SL-DR | 0.695 | 0.043 | 0.82 | 0.99 | 0.09 | 0.089 | 0.703 | 0.053 | 0.82 | 0.99 | 0.084 | 0.084 |
|  | GOAL | 0.698 | 0.089 | 1 | 1 | 0.044 | 0.044 | 0.707 | 0.15 | 1 | 0.98 | 0.079 | 0.079 |
| WoSt  rho=0 | GOALDeR | 0.723 | 0.022 | 0.65 | 1 | 0.035 | 0.042 | 0.712 | 0.033 | 0.83 | 1 | 0.045 | 0.046 |
|  | SL-DR | 0.686 | 0.049 | 0.78 | 0.98 | 0.218 | 0.217 | 0.703 | 0.029 | 0.69 | 1 | 0.058 | 0.058 |
|  | GOAL | 0.708 | 0.087 | 0.95 | 1 | 0.072 | 0.072 | 0.72 | 0.14 | 0.96 | 0.99 | 0.101 | 0.102 |
| SoSt  rho=0.2 | GOALDeR | 0.704 | 0.021 | 0.83 | 1 | 0.03 | 0.03 | 0.707 | 0.032 | 0.85 | 1 | 0.042 | 0.042 |
|  | SL-DR | 0.7 | 0.028 | 0.73 | 1 | 0.05 | 0.05 | 0.709 | 0.031 | 0.63 | 1 | 0.062 | 0.062 |
|  | GOAL | 0.721 | 0.137 | 0.98 | 1 | 0.103 | 0.105 | 0.776 | 0.183 | 0.99 | 0.99 | 0.118 | 0.14 |
| SoWt  rho=0.2 | GOALDeR | 0.689 | 0.025 | 0.83 | 1 | 0.032 | 0.034 | 0.684 | 0.038 | 0.9 | 1 | 0.043 | 0.046 |
|  | SL-DR | 0.714 | 0.048 | 0.74 | 1 | 0.106 | 0.107 | 0.69 | 0.052 | 0.73 | 0.99 | 0.093 | 0.093 |
|  | GOAL | 0.718 | 0.136 | 1 | 0.99 | 0.087 | 0.088 | 0.743 | 0.206 | 0.99 | 0.99 | 0.128 | 0.134 |
| WoSt  rho=0.2 | GOALDeR | 0.699 | 0.021 | 0.88 | 1 | 0.029 | 0.028 | 0.706 | 0.031 | 0.74 | 1 | 0.049 | 0.049 |
|  | SL-DR | 0.699 | 0.035 | 0.73 | 0.99 | 0.056 | 0.056 | 0.705 | 0.04 | 0.67 | 0.99 | 0.079 | 0.079 |
|  | GOAL | 0.723 | 0.125 | 0.99 | 1 | 0.102 | 0.104 | 0.769 | 0.16 | 0.91 | 0.99 | 0.159 | 0.172 |
| SoSt  rho=0.5 | GOALDeR | 0.68 | 0.024 | 0.77 | 1 | 0.034 | 0.039 | 0.663 | 0.031 | 0.59 | 1 | 0.056 | 0.066 |
|  | SL-DR | 0.697 | 0.035 | 0.62 | 1 | 0.128 | 0.128 | 0.658 | 0.032 | 0.45 | 0.99 | 0.176 | 0.18 |
|  | GOAL | 0.735 | 0.183 | 0.95 | 0.89 | 0.158 | 0.161 | 0.836 | 0.221 | 0.88 | 0.87 | 0.209 | 0.248 |
| SoWt  rho=0.5 | GOALDeR | 0.669 | 0.029 | 0.78 | 1 | 0.032 | 0.044 | 0.664 | 0.038 | 0.73 | 1 | 0.057 | 0.068 |
|  | SL-DR | 0.671 | 0.032 | 0.65 | 0.99 | 0.06 | 0.066 | 0.676 | 0.054 | 0.59 | 0.99 | 0.126 | 0.128 |
|  | GOAL | 0.703 | 0.174 | 0.99 | 0.96 | 0.12 | 0.119 | 0.8 | 0.25 | 0.97 | 0.9 | 0.187 | 0.211 |
| WoSt  rho=0.5 | GOALDeR | 0.687 | 0.024 | 0.8 | 1 | 0.034 | 0.037 | 0.683 | 0.031 | 0.74 | 1 | 0.049 | 0.051 |
|  | SL-DR | 0.693 | 0.032 | 0.69 | 1 | 0.051 | 0.051 | 0.68 | 0.026 | 0.5 | 1 | 0.062 | 0.065 |
|  | GOAL | 0.737 | 0.161 | 0.94 | 0.95 | 0.138 | 0.142 | 0.798 | 0.199 | 0.89 | 0.93 | 0.206 | 0.228 |

Notations: DRF =0.7, the parameter of DRF is 0.7; rho=0, rho=0.2, and rho=0.5, there are no, moderate and strong correlations between covariates; emp_std: the empirical standard deviation of the estimates; Est_Std: the mean of standard deviation estimates using sandwich-type estimator (GOAL) or regression of the treatment on pseudo-outcome (GOALDeR and SL-DR); coverage: coverage probability of the 95% confidence interval; RMSE: root mean square error calculated as $RMSE=\sqrt{\frac{1}{B}\sum_{b=1}^{B} \left( \hat{\eta}_{b}-\eta\right)^{2}}$.

**Table S4** Summary statistics of the performance under Scenario 1 with the true parameter of DRF = 0.4

|  |  | **N=500, P=200** | | | | | | **N=200, P=100** | | | | | |
| --- | --- | --- | --- | --- | --- | --- | --- | --- | --- | --- | --- | --- | --- |
|  |  | **DRF** | **Est_**  **Std** | **Est_**  **Coverage** | **Est_**  **Power** | **emp_**  **std** | **RMSE** | **DRF** | **Est_**  **Std** | **Est_**  **Coverage** | **Est_**  **Power** | **emp_**  **std** | **RMSE** |
| SoSt  rho=0 | GOALDeR | 0.409 | 0.024 | 0.8 | 1 | 0.033 | 0.035 | 0.415 | 0.034 | 0.87 | 1 | 0.039 | 0.041 |
|  | SL-DR | 0.401 | 0.037 | 0.79 | 0.96 | 0.088 | 0.087 | 0.424 | 0.039 | 0.64 | 0.99 | 0.127 | 0.128 |
|  | GOAL | 0.412 | 0.1 | 1 | 0.99 | 0.052 | 0.053 | 0.425 | 0.162 | 1 | 0.8 | 0.106 | 0.109 |
| SoWt  rho=0 | GOALDeR | 0.393 | 0.027 | 0.88 | 1 | 0.03 | 0.031 | 0.387 | 0.039 | 0.89 | 1 | 0.045 | 0.047 |
|  | SL-DR | 0.396 | 0.045 | 0.79 | 0.97 | 0.124 | 0.124 | 0.394 | 0.046 | 0.76 | 0.98 | 0.096 | 0.095 |
|  | GOAL | 0.398 | 0.087 | 0.99 | 0.99 | 0.056 | 0.056 | 0.404 | 0.151 | 1 | 0.84 | 0.096 | 0.096 |
| WoSt  rho=0 | GOALDeR | 0.427 | 0.022 | 0.69 | 1 | 0.031 | 0.041 | 0.4 | 0.033 | 0.83 | 1 | 0.049 | 0.049 |
|  | SL-DR | 0.418 | 0.031 | 0.73 | 1 | 0.063 | 0.065 | 0.401 | 0.031 | 0.67 | 0.99 | 0.058 | 0.057 |
|  | GOAL | 0.402 | 0.087 | 0.96 | 0.98 | 0.064 | 0.063 | 0.402 | 0.133 | 0.97 | 0.83 | 0.095 | 0.095 |
| SoSt  rho=0.2 | GOALDeR | 0.4 | 0.021 | 0.81 | 1 | 0.031 | 0.031 | 0.401 | 0.031 | 0.81 | 1 | 0.045 | 0.045 |
|  | SL-DR | 0.403 | 0.033 | 0.59 | 0.98 | 0.065 | 0.064 | 0.42 | 0.042 | 0.67 | 0.99 | 0.118 | 0.12 |
|  | GOAL | 0.403 | 0.148 | 0.99 | 0.8 | 0.117 | 0.116 | 0.466 | 0.185 | 0.97 | 0.77 | 0.132 | 0.147 |
| SoWt  rho=0.2 | GOALDeR | 0.39 | 0.025 | 0.89 | 1 | 0.029 | 0.031 | 0.381 | 0.038 | 0.86 | 1 | 0.05 | 0.053 |
|  | SL-DR | 0.396 | 0.039 | 0.8 | 0.99 | 0.059 | 0.059 | 0.406 | 0.048 | 0.69 | 0.98 | 0.1 | 0.1 |
|  | GOAL | 0.41 | 0.13 | 0.99 | 0.96 | 0.072 | 0.073 | 0.398 | 0.2 | 1 | 0.62 | 0.117 | 0.116 |
| WoSt  rho=0.2 | GOALDeR | 0.402 | 0.021 | 0.88 | 1 | 0.028 | 0.028 | 0.397 | 0.031 | 0.8 | 1 | 0.051 | 0.051 |
|  | SL-DR | 0.391 | 0.033 | 0.78 | 0.98 | 0.055 | 0.056 | 0.369 | 0.039 | 0.61 | 0.96 | 0.124 | 0.127 |
|  | GOAL | 0.41 | 0.122 | 0.99 | 0.89 | 0.098 | 0.098 | 0.468 | 0.159 | 0.95 | 0.87 | 0.116 | 0.134 |
| SoSt  rho=0.5 | GOALDeR | 0.384 | 0.024 | 0.78 | 1 | 0.033 | 0.037 | 0.381 | 0.031 | 0.7 | 1 | 0.054 | 0.056 |
|  | SL-DR | 0.385 | 0.024 | 0.59 | 0.99 | 0.039 | 0.042 | 0.38 | 0.028 | 0.49 | 0.98 | 0.062 | 0.065 |
|  | GOAL | 0.446 | 0.183 | 0.99 | 0.69 | 0.146 | 0.152 | 0.512 | 0.223 | 0.9 | 0.71 | 0.213 | 0.24 |
| SoWt  rho=0.5 | GOALDeR | 0.372 | 0.029 | 0.79 | 1 | 0.035 | 0.044 | 0.354 | 0.039 | 0.69 | 1 | 0.056 | 0.073 |
|  | SL-DR | 0.381 | 0.034 | 0.64 | 0.99 | 0.083 | 0.085 | 0.361 | 0.037 | 0.56 | 0.96 | 0.072 | 0.082 |
|  | GOAL | 0.405 | 0.186 | 0.99 | 0.62 | 0.114 | 0.114 | 0.448 | 0.244 | 0.96 | 0.51 | 0.19 | 0.195 |
| WoSt  rho=0.5 | GOALDeR | 0.384 | 0.024 | 0.78 | 1 | 0.033 | 0.037 | 0.376 | 0.031 | 0.68 | 1 | 0.06 | 0.064 |
|  | SL-DR | 0.385 | 0.024 | 0.71 | 0.99 | 0.035 | 0.038 | 0.37 | 0.032 | 0.45 | 0.99 | 0.067 | 0.073 |
|  | GOAL | 0.445 | 0.16 | 0.98 | 0.82 | 0.136 | 0.143 | 0.476 | 0.225 | 0.94 | 0.6 | 0.203 | 0.216 |

Notations: DRF =0.4, the parameter of DRF is 0.4; rho=0, rho=0.2, and rho=0.5, there are no, moderate and strong correlations between covariates; emp_std: the empirical standard deviation of the estimates; Est_Std: the mean of standard deviation estimates using sandwich-type estimator (GOAL) or regression of the treatment on pseudo-outcome (GOALDeR and SL-DR); coverage: coverage probability of the 95% confidence interval; RMSE: root mean square error calculated as $RMSE=\sqrt{\frac{1}{B}\sum_{b=1}^{B} \left( \hat{\eta}_{b}-\eta\right)^{2}}$.

**Table S5** Summary statistics of the performance under Scenario 2 with the true parameter of DRF = 0.7

|  |  | **N=500, P=200** | | | | | | **N=200, P=100** | | | | | |
| --- | --- | --- | --- | --- | --- | --- | --- | --- | --- | --- | --- | --- | --- |
|  |  | **DRF** | **Est_**  **Std** | **Est_**  **Coverage** | **Est_**  **Power** | **emp_std** | **RMSE** | **DRF** | **Est_**  **Std** | **Est_**  **Coverage** | **Est_**  **Power** | **emp_std** | **RMSE** |
| CoMt  rho=0 | GOALDeR | 0.721 | 0.001 | 0 | 1 | 0.001 | 0.021 | 0.72 | 0.002 | 0 | 1 | 0.002 | 0.02 |
|  | SL-DR | 0.754 | 0.009 | 0.02 | 1 | 0.057 | 0.079 | 0.741 | 0.01 | 0.05 | 1 | 0.075 | 0.085 |
|  | GOAL | 0.724 | 0.011 | 0.44 | 1 | 0.015 | 0.028 | 0.724 | 0.014 | 0.45 | 1 | 0.014 | 0.028 |
| MoCt  rho=0 | GOALDeR | 0.759 | 0.057 | 0.81 | 1 | 0.052 | 0.078 | 0.767 | 0.077 | 0.84 | 1 | 0.08 | 0.104 |
|  | SL-DR | 0.743 | 0.066 | 0.68 | 0.99 | 0.106 | 0.113 | 0.756 | 0.08 | 0.65 | 0.98 | 0.15 | 0.159 |
|  | GOAL | 0.763 | 2.275 | 1 | 0 | 0.169 | 0.179 | 0.816 | 3.146 | 1 | 0 | 0.23 | 0.256 |
| MoMt  rho=0 | GOALDeR | 1.679 | 0.002 | 0 | 1 | 0.002 | 0.979 | 1.68 | 0.004 | 0 | 1 | 0.004 | 0.98 |
|  | SL-DR | 1.877 | 0.04 | 0 | 1 | 0.233 | 1.199 | 1.783 | 0.032 | 0 | 1 | 0.239 | 1.109 |
|  | GOAL | 1.688 | 0.017 | 0 | 1 | 0.018 | 0.988 | 1.689 | 0.022 | 0 | 1 | 0.025 | 0.989 |
| CoMt  rho=0.2 | GOALDeR | 0.722 | 0.001 | 0 | 1 | 0.001 | 0.022 | 0.722 | 0.002 | 0 | 1 | 0.001 | 0.022 |
|  | SL-DR | 0.777 | 0.013 | 0.01 | 1 | 0.072 | 0.105 | 0.758 | 0.011 | 0.01 | 1 | 0.084 | 0.101 |
|  | GOAL | 0.739 | 0.015 | 0.21 | 1 | 0.017 | 0.043 | 0.734 | 0.017 | 0.32 | 1 | 0.016 | 0.037 |
| MoCt  rho=0.2 | GOALDeR | 0.722 | 0.05 | 0.83 | 1 | 0.08 | 0.083 | 0.741 | 0.062 | 0.58 | 1 | 0.142 | 0.147 |
|  | SL-DR | 0.718 | 0.057 | 0.56 | 0.98 | 0.091 | 0.092 | 0.721 | 0.054 | 0.5 | 1 | 0.134 | 0.135 |
|  | GOAL | 0.875 | 2.658 | 1 | 0 | 0.717 | 0.734 | 1.266 | 3.447 | 0.99 | 0.01 | 1.161 | 1.287 |
| MoMt  rho=0.2 | GOALDeR | 1.672 | 0.002 | 0 | 1 | 0.002 | 0.972 | 1.672 | 0.004 | 0 | 1 | 0.003 | 0.972 |
|  | SL-DR | 1.881 | 0.04 | 0 | 1 | 0.243 | 1.206 | 1.795 | 0.034 | 0 | 1 | 0.264 | 1.126 |
|  | GOAL | 1.689 | 0.016 | 0 | 1 | 0.02 | 0.989 | 1.694 | 0.02 | 0 | 1 | 0.026 | 0.994 |
| CoMt  rho=0.5 | GOALDeR | 0.723 | 0.001 | 0 | 1 | 0.001 | 0.023 | 0.723 | 0.002 | 0 | 1 | 0.002 | 0.023 |
|  | SL-DR | 0.843 | 0.023 | 0 | 1 | 0.117 | 0.185 | 0.767 | 0.011 | 0 | 1 | 0.111 | 0.129 |
|  | GOAL | 0.749 | 0.019 | 0.23 | 1 | 0.031 | 0.058 | 0.747 | 0.023 | 0.24 | 1 | 0.041 | 0.062 |
| MoCt  rho=0.5 | GOALDeR | 0.679 | 0.048 | 0.49 | 1 | 0.123 | 0.125 | 0.646 | 0.06 | 0.48 | 0.99 | 0.186 | 0.193 |
|  | SL-DR | 0.737 | 0.042 | 0.33 | 0.99 | 0.132 | 0.137 | 0.694 | 0.064 | 0.36 | 0.98 | 0.225 | 0.224 |
|  | GOAL | 1.406 | 3.358 | 1 | 0.01 | 1.802 | 1.927 | 2.884 | 4.623 | 0.85 | 0.17 | 4.901 | 5.343 |
| MoMt  rho=0.5 | GOALDeR | 1.665 | 0.002 | 0 | 1 | 0.002 | 0.965 | 1.665 | 0.003 | 0 | 1 | 0.003 | 0.965 |
|  | SL-DR | 1.953 | 0.052 | 0 | 1 | 0.35 | 1.3 | 1.878 | 0.056 | 0 | 1 | 0.385 | 1.238 |
|  | GOAL | 1.689 | 0.017 | 0 | 1 | 0.024 | 0.989 | 1.7 | 0.021 | 0 | 1 | 0.026 | 1 |

Notations: DRF =0.7, the parameter of DRF is 0.7; rho=0, rho=0.2, and rho=0.5, there are no, moderate and strong correlations between covariates; emp_std: the empirical standard deviation of the estimates; Est_Std: the mean of standard deviation estimates using sandwich-type estimator (GOAL) or regression of the treatment on pseudo-outcome (GOALDeR and SL-DR); coverage: coverage probability of the 95% confidence interval; RMSE: root mean square error calculated as $RMSE=\sqrt{\frac{1}{B}\sum_{b=1}^{B} \left( \hat{\eta}_{b}-\eta\right)^{2}}$.

**Table S6** Summary statistics of the performance under Scenario 2 with the true parameter of DRF = 0.4

|  |  | **N=500, P=200** | | | | | | **N=200, P=100** | | | | | |
| --- | --- | --- | --- | --- | --- | --- | --- | --- | --- | --- | --- | --- | --- |
|  |  | **DRF** | **Est_**  **Std** | **Est_**  **Coverage** | **Est_**  **Power** | **emp_std** | **RMSE** | **DRF** | **Est_**  **Std** | **Est_**  **Coverage** | **Est_**  **Power** | **emp_std** | **RMSE** |
| CoMt  rho=0 | GOALDeR | 0.424 | 0.001 | 0 | 1 | 0.001 | 0.024 | 0.423 | 0.001 | 0 | 1 | 0.002 | 0.023 |
|  | SL-DR | 0.428 | 0.006 | 0.13 | 1 | 0.028 | 0.039 | 0.427 | 0.005 | 0.03 | 1 | 0.047 | 0.054 |
|  | GOAL | 0.424 | 0.012 | 0.34 | 1 | 0.014 | 0.028 | 0.423 | 0.014 | 0.48 | 1 | 0.016 | 0.028 |
| MoCt  rho=0 | GOALDeR | 0.445 | 0.058 | 0.93 | 1 | 0.046 | 0.064 | 0.482 | 0.078 | 0.81 | 1 | 0.084 | 0.117 |
|  | SL-DR | 0.422 | 0.075 | 0.77 | 0.92 | 0.131 | 0.132 | 0.445 | 0.075 | 0.56 | 0.95 | 0.157 | 0.162 |
|  | GOAL | 0.44 | 2.292 | 1 | 0 | 0.177 | 0.181 | 0.565 | 3.067 | 1 | 0 | 0.298 | 0.339 |
| MoMt  rho=0 | GOALDeR | 1.388 | 0.002 | 0 | 1 | 0.002 | 0.988 | 1.388 | 0.003 | 0 | 1 | 0.003 | 0.988 |
|  | SL-DR | 1.486 | 0.023 | 0 | 1 | 0.123 | 1.093 | 1.432 | 0.015 | 0 | 1 | 0.115 | 1.039 |
|  | GOAL | 1.392 | 0.019 | 0 | 1 | 0.02 | 0.992 | 1.394 | 0.019 | 0 | 1 | 0.022 | 0.994 |
| CoMt  rho=0.2 | GOALDeR | 0.424 | 0.001 | 0 | 1 | 0.001 | 0.024 | 0.423 | 0.001 | 0 | 1 | 0.002 | 0.023 |
|  | SL-DR | 0.429 | 0.005 | 0.09 | 1 | 0.023 | 0.037 | 0.427 | 0.003 | 0.06 | 1 | 0.015 | 0.031 |
|  | GOAL | 0.437 | 0.016 | 0.28 | 1 | 0.017 | 0.041 | 0.44 | 0.015 | 0.18 | 1 | 0.015 | 0.042 |
| MoCt  rho=0.2 | GOALDeR | 0.431 | 0.049 | 0.69 | 1 | 0.079 | 0.085 | 0.435 | 0.061 | 0.7 | 1 | 0.12 | 0.125 |
|  | SL-DR | 0.428 | 0.054 | 0.66 | 0.98 | 0.101 | 0.105 | 0.439 | 0.052 | 0.48 | 0.96 | 0.153 | 0.157 |
|  | GOAL | 0.474 | 2.673 | 1 | 0 | 0.598 | 0.599 | 0.729 | 3.717 | 1 | 0 | 1.182 | 1.221 |
| MoMt  rho=0.2 | GOALDeR | 1.381 | 0.002 | 0 | 1 | 0.002 | 0.981 | 1.381 | 0.003 | 0 | 1 | 0.003 | 0.981 |
|  | SL-DR | 1.497 | 0.024 | 0 | 1 | 0.136 | 1.105 | 1.449 | 0.019 | 0 | 1 | 0.128 | 1.057 |
|  | GOAL | 1.39 | 0.014 | 0 | 1 | 0.016 | 0.99 | 1.397 | 0.03 | 0.01 | 0.99 | 0.084 | 1 |
| CoMt  rho=0.5 | GOALDeR | 0.423 | 0.001 | 0 | 1 | 0.001 | 0.023 | 0.423 | 0.001 | 0 | 1 | 0.002 | 0.023 |
|  | SL-DR | 0.446 | 0.008 | 0.08 | 1 | 0.045 | 0.064 | 0.44 | 0.006 | 0.01 | 1 | 0.041 | 0.057 |
|  | GOAL | 0.452 | 0.016 | 0.14 | 1 | 0.025 | 0.058 | 0.446 | 0.024 | 0.31 | 0.99 | 0.033 | 0.057 |
| MoCt  rho=0.5 | GOALDeR | 0.363 | 0.049 | 0.46 | 1 | 0.111 | 0.116 | 0.317 | 0.062 | 0.39 | 0.83 | 0.172 | 0.19 |
|  | SL-DR | 0.449 | 0.039 | 0.37 | 0.99 | 0.122 | 0.131 | 0.439 | 0.043 | 0.32 | 0.97 | 0.192 | 0.195 |
|  | GOAL | 1.343 | 4.044 | 1 | 0 | 3.04 | 3.169 | 2.516 | 5.118 | 0.84 | 0.19 | 4.74 | 5.169 |
| MoMt  rho=0.5 | GOALDeR | 1.374 | 0.002 | 0 | 1 | 0.002 | 0.974 | 1.374 | 0.003 | 0 | 1 | 0.003 | 0.974 |
|  | SL-DR | 1.581 | 0.04 | 0 | 1 | 0.238 | 1.204 | 1.476 | 0.029 | 0 | 1 | 0.22 | 1.098 |
|  | GOAL | 1.391 | 0.016 | 0 | 1 | 0.022 | 0.992 | 1.395 | 0.022 | 0 | 1 | 0.035 | 0.996 |

Notations: DRF =0.4, the parameter of DRF is 0.4; rho=0, rho=0.2, and rho=0.5, there are no, moderate and strong correlations between covariates; emp_std: the empirical standard deviation of the estimates; Est_Std: the mean of standard deviation estimates using sandwich-type estimator (GOAL) or regression of the treatment on pseudo-outcome (GOALDeR and SL-DR); coverage: coverage probability of the 95% confidence interval; RMSE: root mean square error calculated as $RMSE=\sqrt{\frac{1}{B}\sum_{b=1}^{B} \left( \hat{\eta}_{b}-\eta\right)^{2}}$.
